# Methodological development of tools to measure how women are treated during facility-based childbirth in four countries: labor observation and community survey

**DOI:** 10.1186/s12874-018-0603-x

**Published:** 2018-11-15

**Authors:** Meghan A. Bohren, Joshua P. Vogel, Bukola Fawole, Ernest T. Maya, Thae Maung Maung, Mamadou Diouldé Baldé, Agnes A. Oyeniran, Modupe Ogunlade, Kwame Adu-Bonsaffoh, Nwe Oo Mon, Boubacar Alpha Diallo, Abou Bangoura, Richard Adanu, Sihem Landoulsi, A. Metin Gülmezoglu, Özge Tunçalp

**Affiliations:** 1 UNDP/UNFPA/UNICEF/WHO/World Bank Special Programme of Research, Development and Research Training in Human Reproduction, Department of Reproductive Health and Research, World Health Organization, 1211 Geneva, Switzerland; 2 Gender and Women’s Health Unit, Centre for Health Equity, Melbourne School of Population and Global Health, The University of Melbourne, Carlton, VIC 3053 Australia; 3 Department of Obstetrics and Gynecology, National Institute of Maternal and Child Health, College of Medicine, University of Ibadan, Ibadan, Nigeria; 4 School of Public Health, University of Ghana, Accra, Ghana; 5 Department of Medical Research, Yangon, Myanmar; 6 Cellule de Recherche en Santé de la Reproduction en Guinée (CERREGUI), University National Hospital-Donka, Conakry, Guinea; 7 Faculté de Médecine, Pharmacie et Odontostomatologie, Université G.A. Nasser de Conakry, Conakry, Guinea; 8 Department of Health Promotion and Education, Faculty of Public Health, College of Medicine, University of Ibadan, Ibadan, Nigeria; 9 Department of Obstetrics and Gynecology, School of Medicine and Dentistry, University of Ghana, Accra, Ghana; 10 Département de sociologie, Université Sonfonia, Conakry, Guinea

**Keywords:** Maternal health, Obstetric delivery, Childbirth, Quality of care, Mistreatment, Disrespect and abuse, Nigeria, Ghana, Guinea, Myanmar

## Abstract

**Background:**

Efforts to improve maternal health are increasingly focused on improving the quality of care provided to women at health facilities, including the promotion of respectful care and eliminating mistreatment of women during childbirth. A WHO-led multi-country research project aims to develop and validate two tools (labor observation and community survey) to measure how women are treated during facility-based childbirth. This paper describes the development process for these measurement tools, and how they were implemented in a multi-country study (Ghana, Guinea, Myanmar and Nigeria).

**Methods:**

An iterative mixed-methods approach was used to develop two measurement tools. Methodological development was conducted in four steps: (1) initial tool development; (2) validity testing, item adjustment and piloting of paper-based tools; (3) conversion to digital, tablet-based tools; and (4) data collection and analysis. These steps included systematic reviews, primary qualitative research, mapping of existing tools, item consolidation, peer review by key stakeholders and piloting.

**Results:**

The development, structure, administration format, and implementation of the labor observation and community survey tools are described. For the labor observations, a total of 2016 women participated: 408 in Nigeria, 682 in Guinea, and 926 in Ghana. For the community survey, a total of 2672 women participated: 561 in Nigeria, 644 in Guinea, 836 in Ghana, and 631 in Myanmar. Of the 2016 women who participated in the labor observations, 1536 women (76.2%) also participated in the community survey and have linked data: 779 in Ghana, 425 in Guinea, and 332 in Nigeria.

**Conclusions:**

An important step to improve the quality of maternity care is to understand the magnitude and burden of mistreatment across contexts. Researchers and healthcare providers in maternal health are encouraged to use and implement these tools, to inform the development of more women-centered, respectful maternity healthcare services. By measuring the prevalence of mistreatment of women during childbirth, we will be able to design and implement programs and policies to transform maternity services.

**Electronic supplementary material:**

The online version of this article (10.1186/s12874-018-0603-x) contains supplementary material, which is available to authorized users.

## Background

Worldwide, an estimated 303,000 maternal deaths occurred in 2015, 99% of which were in low- and middle-income countries (LMIC) [1]. Efforts to improve maternal health have historically focused on increasing the rate and coverage of antenatal care, skilled birth attendance, and births occurring in health facilities. However, there is a growing focus on the importance of ensuring good quality maternal healthcare to improve health outcomes. In 2015, the World Health Organization (WHO) proposed a global vision where ‘every pregnant woman and newborn receives quality care throughout pregnancy, childbirth and the postnatal period’ [2], and highlighted the importance of considering both how care is provided by health workers within health systems, and how care is experienced by users (particularly pregnant women and their families). The WHO framework for quality of care explicitly identifies effective communication, respect, dignity, and emotional support as key domains of quality to improve women’s and newborns’ experiences of care [2].

Quality of care has been further emphasized by the 2018 “WHO recommendations on intrapartum care for a positive childbirth experience,” that extend beyond the prevention of mortality and morbidity to encompass a woman-centered, rights-based approach to optimizing health and well-being for women and their babies [3, 4]. The new recommendations articulate the overarching importance of respectful maternity care, and the need for staff and health services to create enabling maternity care environments that encourage a woman’s sense of control and their involvement in decision-making [3]. It also contains specific recommendations on respectful care policies, labor companionship and effective communication. These recommendations reflect a growing body of literature demonstrating that women may experience abusive, neglectful or disrespectful care during labor and childbirth in healthcare facilities [5]. These negative experiences may inhibit women from attending health facilities for childbirth, or using them in the future [6].

In 2010, Bowser and Hill published a landscape analysis outlining the issue of disrespectful and abusive care women experienced during childbirth in health facilities [7]. Since then, WHO published a statement in 2014 calling for the “Prevention and elimination of disrespect and abuse during childbirth,” which has been endorsed by over 90 organizations worldwide [8]. A 2015 publication by Bohren and colleagues proposed a typology for the mistreatment of women during childbirth, based on a mixed-methods systematic review that included evidence from 65 studies conducted in 34 countries [5]. This work proposed that the mistreatment of women during childbirth includes physical and verbal abuse, discrimination, neglect, and health systems constraints [5], which can amount to human rights violations [9]. The mistreatment of women during childbirth is a multidimensional issue that requires understanding of complex social norms related to gender equality, power dynamics, and clinical hierarchies [10, 11].

### Mistreatment within the context of women’s health globally

The Sustainable Development Goal (SDG) era presents the global community with an exciting opportunity to improve health, well-being and equality for all women. SDG 3 includes targets to continue the reduction of the maternal mortality ratio and ensure universal access to sexual and reproductive health care services [12]. Relatedly, SDG 5 targets include ending all forms of discrimination, violence, and harmful practices against women and girls [12]. Similarly, the Global Strategy for Women’s, Children’s and Adolescents’ Health was launched by the United Nations in 2015 and outlines an ambitious approach to end preventable deaths (survive), ensure health and well-being (thrive), and expand enabling environments (transform), while leaving no one behind [13]. The Global Strategy seeks to ensure that all women not only survive childbirth and any complications if they arise, but that all women can reach their full potential for life and health. These goals promote a global landscape where reducing health and gender inequalities, and promoting positive healthcare experiences are paramount to transform our world. Eliminating all forms of mistreatment of women during childbirth is a critical pathway to achieve these goals and transform society.

### Measuring the mistreatment of women during childbirth

Bowser and Hill’s landscape analysis spurred the conduct of several research projects on measuring disrespect and abuse during childbirth, including studies in Kenya, Tanzania, Nigeria, and Ethiopia [14–25]. These studies were informed by the definitions and categories proposed by Bowser and Hill. However data collection methods and tools varied, including direct observations of labor and childbirth [21], facility exit interviews with women [14, 18, 24, 26], and interviews with women during the postpartum period [24–26]. Prevalence estimates vary widely between individual studies (12.2 to 98% across studies conducted in LMIC settings), which is at least partly due to these methodological differences. Sando and colleagues reviewed the methods used in five prevalence studies, identifying several key differences between measurement approaches [27]. A standard approach to defining and measuring mistreatment of women during childbirth, through the development of validated measurement tools, would therefore permit standardized comparisons of prevalence data across settings and over time.

### Rationale for terminology

Different terminologies have been used to describe the phenomenon of mistreatment during childbirth, including obstetric violence, disrespect and abuse, and respectful maternity care. For the purposes of our study, we have used the term “mistreatment of women during childbirth” to convey the phenomenon of interest in a way that places the woman at the center of the experience, e.g. to promote a woman-centered measurement approach. This terminology uses language that does not assign blame, which has helped us to form multidisciplinary teams of women, researchers, midwives, nurses, doctors and healthcare administrators, and reduced the risk of alienating certain groups by passing judgment through vocabulary. Furthermore, we believe that this terminology acknowledges that mistreatment may occur either intentionally or non-intentionally, may result from shortcomings in the health system rather than malicious intent, and may be experienced either at an intrapersonal level between a woman and a healthcare provider or staff, or at a more nuanced level during a woman’s interactions with the health system and infrastructure [28].

### The WHO multi-country study: “How women are treated during facility-based childbirth”

In November 2013, a technical consultation of international experts recommended that WHO initiate advocacy and research activities to develop and validate prevalence measurement tools on the mistreatment of women during childbirth that would provide comparable data of the burden of mistreatment across settings. In 2014 WHO initiated a multi-country research study entitled “How women are treated during facility -based childbirth: development and validation of measurement tools in four countries” [29]. The primary objectives of this study were to develop an evidence-based definition and associated set of identification criteria for the mistreatment of women during childbirth in facilities, and two tools to measure this phenomenon. A two-phased, mixed-methods study design was used to develop identification criteria and tools for measuring mistreatment in facilities, and understand influencing factors in Ghana, Guinea, Myanmar and Nigeria. Phase 1 was a formative phase comprised of a mixed-methods systematic review on mistreatment [5], a qualitative evidence synthesis on respectful maternity care [30], and primary qualitative research (focus group discussions, in-depth interviews) in four countries [10, 29, 31–34]. Findings from Phase 1 informed the measurement component (Phase 2), which used direct observations of labor and childbirth in health facilities, and follow-up community-based surveys with postpartum women to measure mistreatment during childbirth. This paper describes the development of the two measurement tools and how they were implemented in the study sites. Providing this detailed description can help users to understand the robust and systematic development process that was used, and can help inform tool development in related areas.

## Methods

**Fig. 1:**
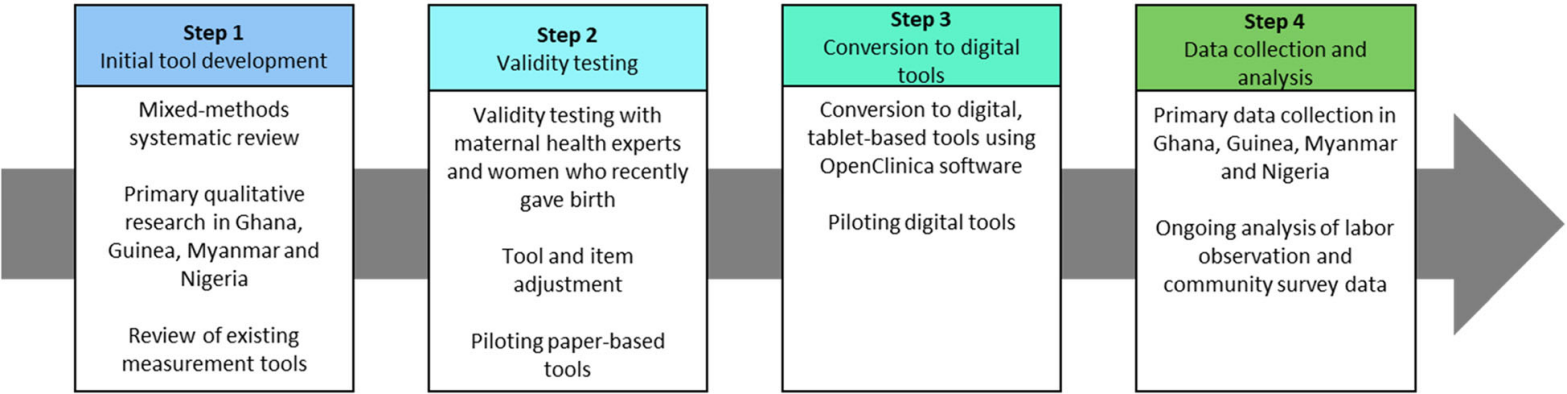
Visual depiction of the tool development process

An iterative mixed-methods approach was used to develop two measurement tools: direct observations of labor and childbirth, and a follow-up community-based survey with women. Methodological development was conducted in four steps (Fig. 1): (1) initial tool development; (2) validity testing, item adjustment and piloting of paper-based tools; (3) conversion to digital, tablet-based tools; and (4) data collection. Several research methods were employed, including systematic reviews, primary qualitative research, mapping of existing tools, item consolidation, peer review by key stakeholders and piloting, and are described in the following sections.

### Step 1: Initial tool development (June 2014 to December 2015)

The overall aim of these tools is to be discriminative; that is to distinguish women who experience mistreatment during childbirth, from those who do not. The multi-country research team discussed and agreed on desired characteristics of the measurement tools, which informed the study design (Table 1).

**Table 1:** Desired characteristics of the study design and measurement tools

Phenomena of interest	Occurrence of mistreatment of women during labor, childbirth, and immediate postpartum period in health facilities
Target population	Women giving birth in participating health facilities in study countries
Time period of interest	From admission to health facility for childbirth, until 2 h postpartum in the health facility or discharge, whichever happens first.
Administration format	Tool #1 –direct observation of women during labor, childbirth, and immediate postpartum Tool #2 – an interviewer-administered survey of postpartum women’s self-reported experiences of how they were treated during childbirth in a health facility conducted several weeks postpartum

#### Identification of domains and items: Evidence synthesis and formative research

The evidence-based typology from the systematic review by Bohren and colleagues provided the initial tool structure and domains [5]. This typology was further informed by findings from the primary qualitative research in four countries (Ghana, Guinea, Myanmar and Nigeria) with women, providers and administrators [10, 31–34], to provide illustrative quotes of specific themes and sub-themes to inform development of tool items (i.e. questions). The research team developed an initial list of potential items through discussion and consensus.

#### Identification of domains and items: Review of existing measurement tools

Tools and specific items have been developed and applied in several recent studies that relate to aspects of how women are treated during childbirth. We identified relevant existing tools through a) measurement studies identified via the mixed-methods systematic review [5], and b) contacting key stakeholders working in maternal and perinatal health research and quality of maternity care (including those working on issues of respect, dignity and mistreatment during childbirth) to share any existing measurement tools. In some instances, tools related only to a subset of domains/items within the typology.

Through this process we identified 36 tools [14, 18, 21–25, 35–63]. All identified tools were reviewed, and relevant items were mapped to the corresponding domains of mistreatment. For some domains (such as consenting for vaginal examinations), there were no existing items, or existing items were unsuitable for use in this study. Resulting from this process was a systematic mapping of existing items against the domains of mistreatment identified in the systematic review. The research team then used a consensus process to develop two draft tools based on available items. In some domains (such as types of physical and verbal mistreatment), item reduction or consolidation was required. The labor observation tool and community survey tool were harmonized to the extent possible on a per-domain and per-item basis, to allow comparisons between data collected using the two tools.

### Step 2: Validity testing, item adjustment and piloting of paper-based tools (January to April 2016)

During this step, we aimed to optimize the two tools for:

1. Content validity: to assess if the proposed tools were measuring all aspects of the construct of a woman’s experience of mistreatment, and to identify any additional items that should be considered for inclusion [64]; and
2. Understandability: To ensure the clarity of wording, likelihood that participants and users could answer the questions, and a user-friendly layout and style

#### Validity testing with maternal health experts

We facilitated a meeting with seven global maternal health content experts to review draft versions of both tools. Using a structured approach, these experts were asked to comment on how relevant each item is to the construct it is designed to measure. Experts were also asked to comment on item clarity and conciseness, as well as suggest items that may have been missed. Field notes were taken during the discussion to capture key points, and feedback was incorporated into the tool structure and contents. Key revisions from this step centered around prioritizing items related to maternal and newborn health outcomes, such as which items were feasible and reliable to ask a woman during the community survey, and which items were feasible and reliable to have a non-clinical research assistant assess during the labor observations. Furthermore, the expert group and research team discussed in detail how to document instances of mistreatment during the labor observations, focusing on whether the research priority was to document either (a) the number of times a specific type of mistreatment occurred per women (potential to record recurring events); or (b) whether a specific type of mistreatment occurred or not (record the first event only). Ultimately, there was consensus to document recurring events, in order to better understand the magnitude of mistreatment occurring and to have a more complete documentation of a woman’s childbirth experience.

#### Validity testing with women who recently gave birth in Nigeria

The phenomenon of interest relates to women’s experiences during childbirth in health facilities; therefore, we considered it important to engage women in the development of the community survey tool and ensure proposed items were understandable and considered important to women. The community survey tool was reviewed by two groups of five women from Nigeria (country of the development sample) who recently gave birth. This was done in two face-to-face group discussions, facilitated by two experienced female researchers from the Nigeria team (AAO and MO). For each item, women were asked to provide comments on clarity of wording, understandability and perceived value of the question. A simple scoring system was used for the women to rank the level of importance of the question to themselves and “women like them” in their communities. Field notes were taken during the discussion to capture key points, and a short report was developed to inform the research team. Key revisions from this activity centered around ensuring the language of each question in the community survey was understandable to women, and a better understanding the importance of each item to women. Based on the scoring, all items were included in the revised version of the tools, as women considered the questions to be of importance.

#### Tool and item adjustment

Based on the findings from the content validity testing with maternal health experts and women in the study setting, both tools were revised. We eliminated items of low relevance, or merged items that conveyed similar concepts. Several items were added or adjusted based on feedback, such as integrating newborn care practices into the labor observation tool, and ensuring that maternal health outcome items were articulated in a way that a non-clinician could understand. Final decisions regarding tool structure and items were made through research team consensus.

#### Piloting paper-based tools

The revised tools were formally piloted in one study site in Nigeria. Two female researchers from the Nigeria team (AAO and MO) conducted direct observations of a convenience sample of twenty consenting women throughout labor, childbirth, and the immediate postpartum period. The community survey was piloted separately with a group of ten women who recently gave birth. Feedback from Nigerian research team on tool implementation informed further revision and finalization of the tools, and informed development of the study manual of operations. Piloting the paper tools also helped the research team to identify design considerations for the digital forms, such as how to structure the digital forms according to the time point when the form would be completed (e.g.: at admission, throughout labor, or after childbirth), and whether the form would be completed once (labor observation tool (LOT)-Admission, LOT-Childbirth, community survey screening and community survey) or multiple times (LOT-Incident report).

This step resulted in the final draft of the paper-based tools in English. The research team then collaborated to translate the tools into the languages used in the study contexts. In Nigeria and Ghana, the labor observation tool was in English only, as it was expected that all research assistants would speak English, and no verbal interaction with research participants was necessary to complete the tool. In Guinea, the labor observation tool was translated into French. In contrast, the community survey tool involved interaction with research participants, so local language translation was needed. In Nigeria, the tool was translated into Yoruba; in Guinea, the tool was translated into French, Malinke, Poular, and Soussou; in Ghana, the tool was translated into Twi; and in Myanmar, the tool was translated into Burmese.

### Step 3: Conversion to digital, tablet-based tools and pilot testing (April to august 2016)

Digital versions of the tools were created using the OpenClinica Participate software (OpenClinica open source software, version 3.1, Waltham, MA, USA). This platform met specific study requirements, including complex form structure (e.g.: forms with repeating/multiple or non-repeating/single submission), backend processing for data collection and submission in areas with poor 3G connectivity, offer different language and alphabet requirements (eight languages across four study sites), and maximizing ease of use for data collectors. A low-cost Android tablet, locked for all purposes other than data collection, was used in all sites. Tablet-based forms were piloted in all four study sites by all data collectors, during data collection training workshops. Piloting the forms during the workshops allowed the research team to develop and test responses to scenarios that may arise during data collection, such as handover of labor observations between research assistants (in case the woman did not give birth by the end of the research assistant’s shift), as well as familiarizing the research assistants with the research environment. Only minor revisions around local language translations were made during this step.

### Step 4: Data collection and analysis (September 2016 to February 2018)

WHO staff and local principal investigators conducted dedicated training workshops in each study site for research coordinators, data collectors and other research team members including a midwife from each study site. Each study site had a research coordinator who was also an obstetrician currently practicing at that site. Due to the sensitive nature of this study, all data collectors were female. Most data collectors were public health or social work graduates, and none had a clinical background (such as nursing, midwifery, medicine) to minimize bias. Workshops included: (1) an overview of the study and study design; (2) dissemination of results from qualitative formative research; (3) review of the study manual of operations; (4) piloting tablet-based forms; and (5) developing an implementation plan.

As the development sample, the tools were initially implemented in Nigeria (September 2016 to February 2017). The main revision after this phase was to the structure of the module related to the care around labor and childbirth. The tool initially had an additional form to complete related to inpatient care (pain relief, labor companionship, fluids, mobilization, unreasonable demands, fee structures and neglect). The revised version of the forms implemented in Ghana and Guinea incorporated this form and all questions into the form “Labor observation childbirth, interventions and discharge,” in order to improve efficiency of data collection. No changes were made to the community survey tool after implementation in Nigeria.

Data collection for the validation sample (Ghana, Guinea, Myanmar) was completed from July 2017 to February 2018. Issues identified during training workshops resulted in minor revisions to tablet-based forms (e.g.: ensuring local language translations were accurate and understandable). These revisions were made to the tablet-based forms, and the tablets were reprogrammed to activate for use in the study environment. Labor observations were not conducted in Myanmar, as it was not contextually appropriate for nonclinical researchers to be present on the labor wards.

### Ethical approvals

This study was approved by the World Health Organization Ethical Review Committee (protocol: A65880) and the World Health Organization Human Reproduction Programme (HRP) Review Panel on Research Projects. This study was also approved by in-country ethical committees in: Guinea [le comité national d’éthique pour la recherche en santé]; Nigeria [Federal Capital Territory Health Research Ethics Committee; Research Ethical Review Committee, Oyo State; and State Health Research Ethics Committee of Ondo State]; Ghana [Ethical Review Committee of the Ghana Health Service; Ethical and Protocol Review Committee of the College of Health Sciences, University of Ghana]; and Myanmar [Ethics Review Committee, Department of Medical Research] (full details in Declarations section).

## Results

Data were collected from September 2016 to February 2017 in Nigeria, and from July 2017 to February 2018 in Guinea, Ghana and Myanmar. This section outlines the structure, administration format and implementation of the labor observation and community survey tools in Ghana, Guinea, Nigeria and Myanmar. For each tool, the following aspects are described: an overview of study procedures and workflow, structure and formatting of forms, and implementation of the tool in the study context. Additional study forms for use during implementation are described, including the screening logs, data submission logs, and data collection discrepancy report. The final section describes linking participant data between the labor observations and community surveys.

### Labor observation tool (LOT)

**Fig. 2:**
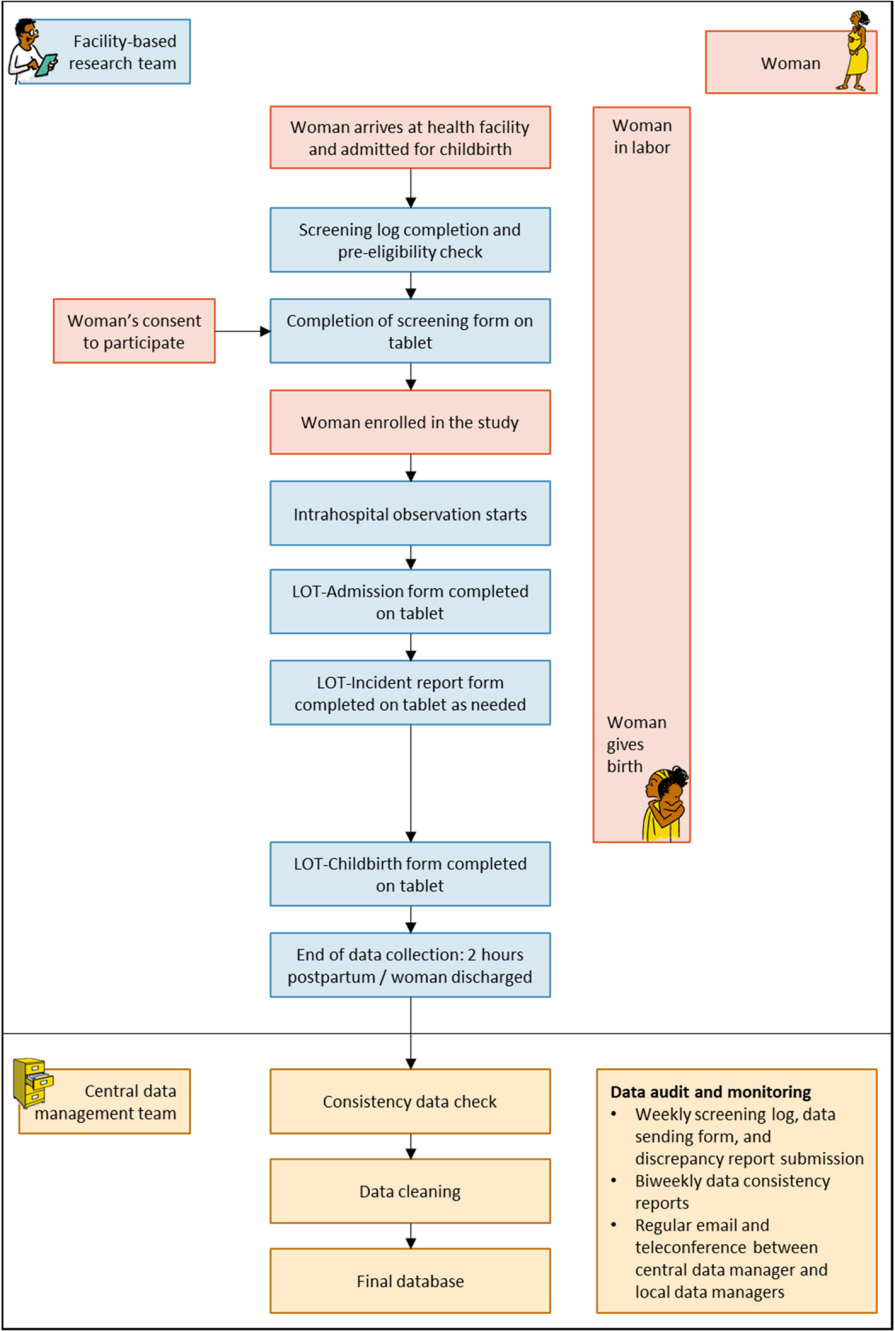
Study procedure and workflow for the labor observations. All images developed by the research team

Potential study participants for the labor observation component were consenting women who were giving birth in study facilities. Pregnant women in established labor (as per the treating clinician’s assessment) who presented to participating facilities during the study period were approached to participate. Eligible women who consented to participate were recruited in the study. Women were then continuously observed from the time of recruitment (at admission for childbirth), through labor and childbirth, until two hours postpartum or discharge (whichever happened first). One research assistant observed only one woman at a given time. Data collectors were instructed to observe women in a quiet, unobtrusive manner and not to contribute to the provision of care. Women were observed continuously even if they were moved between wards or rooms, for example moving from the labor ward to delivery ward (except on the rare occasion that the provision of emergency clinical care prevented observation). Figure 2 depicts the study procedure and workflow for the labor observations.

**Fig. 3:**
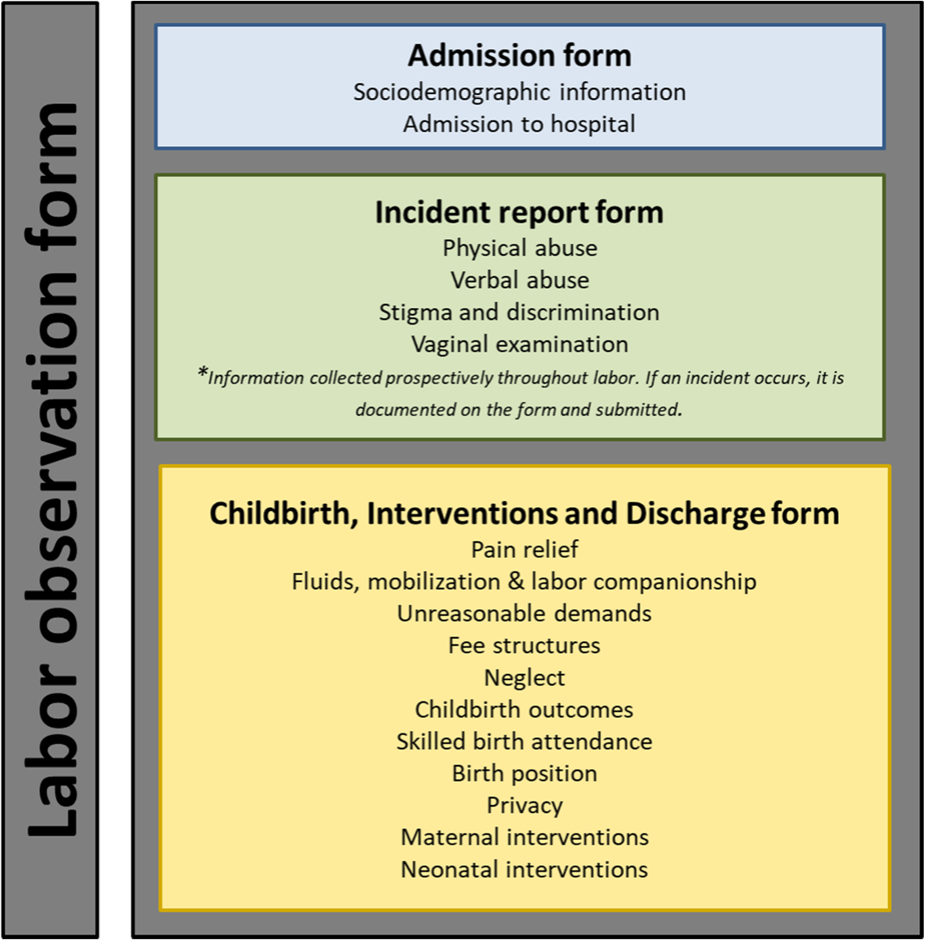
Visual depiction of the structure of the labor observation tool

The tablet-based labor observation tool was used for data collection, available in full in Additional file 1: Labor observation tool. The labor observation tool is comprised of three forms: (1) admission form; (2) incident report form; and (3) childbirth, interventions and discharge form. Figure 3 visually depicts the structure of the labor observation tool.

#### Labor observation tool admission form

The first form completed was the labor observation tool-admission (LOT-Admission) form. This form was completed immediately after the woman was recruited in the study, and was completed only once for all women. This form captures screening questions, and sociodemographic information about the woman, such as her age, education, marital status, and obstetric history.

#### Labor observation tool incident report form

The second form to complete was the labor observation tool-incident report (LOT-Incident Report) form. This form was completed if, and only if, one of the following events occurred: physical abuse, verbal abuse, stigma and discrimination, or a vaginal examination. If one of these incidents occurred, then the form was completed and submitted immediately. This form could have been completed and submitted multiple times, in case of multiple instances of physical abuse, verbal abuse, stigma and discrimination, or vaginal examination (e.g.: repeating form to capture more than one event), or never, in case none of these instances occurred. For instances of physical or verbal abuse, or stigma and discrimination, this form captured information about the timing of the incident (intrapartum or postpartum), the time the incident occurred (00:00–23:59), and who did it (doctor, midwife, nurse, trainee, non-clinical staff, family member or companion of the woman, unknown; possible for multiple people to be involved).

For instances of vaginal examinations, this form captured information about whether the exchange of information, consent, privacy, and confidentiality was observed or not. Because multiple vaginal examinations can occur throughout a woman’s labor, vaginal examinations were reported as “incidents”, so that information could be recorded about multiple events.

#### Labor observation tool childbirth, interventions and discharge form

This form was completed at the end of the period of observation (one per woman). This form captured information about what events transpired throughout the woman’s labor and childbirth period, including pain relief, mobilization, fluids, labor companionship, demands from healthcare providers, fees, neglect, the childbirth outcome, status of the baby, privacy during childbirth, availability of beds, maternal interventions and informed consent, newborn interventions, referral/discharge, and outcome of observation.

#### Implementation of labor observation tool

Overall, 2806 women were screened for the labor observations, and 2019 women (72.0%) were eligible to participate. From this, 2016 women (99.9%) were observed: 408 from Nigeria, 682 from Guinea, and 926 from Ghana. Women who were eligible for participation but were not observed (3 women, 0.1%) were excluded because they did not provide consent. As reported above, labor observations were not conducted in Myanmar, as it was not contextually appropriate for nonclinical researchers to be present on the labor wards.

### Community survey tool (CST)

**Fig. 4:**
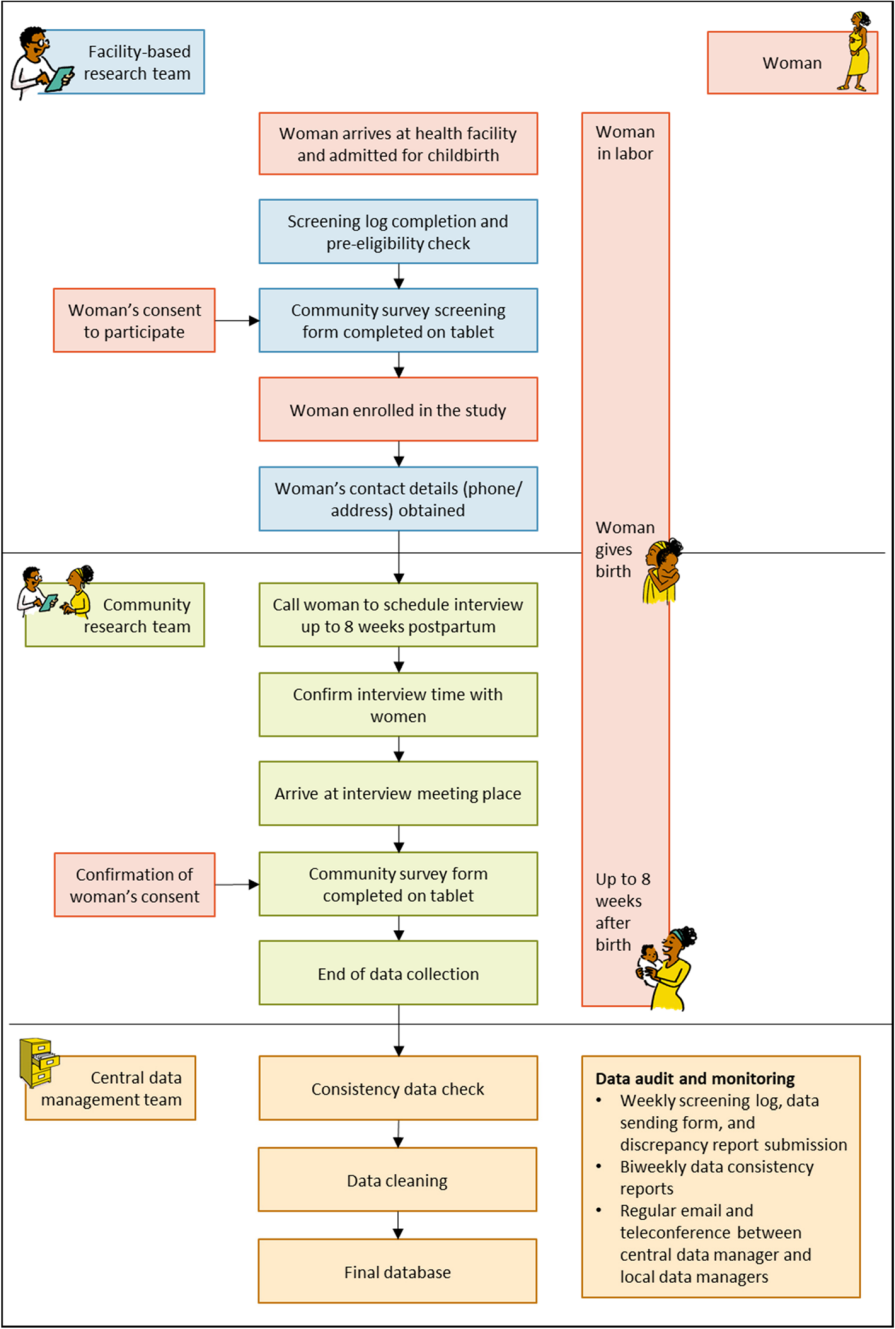
Study procedure and workflow for the community survey. All images developed by the research team

Potential study participants for the community survey component were women who were giving birth in the study facilities and were available for a follow up interview up to eight weeks postpartum. Pregnant women in established labor who presented to participating facilities during the study period were approached to participate. Eligible women who consented to participate were recruited in the study, and contact information was obtained to schedule an interview. Figure 4 depicts the study procedure and workflow for the community survey.

During the survey, the tablet-based community survey tool was used for data collection, available in full in Additional file 2: Community survey tool. The community survey tool is comprised of two forms: (1) community survey screening form; and (2) community survey form.

#### Community survey screening form

The first form to complete was the community survey screening form. This form was completed to assess the woman’s eligibility to participate in the study, and was completed only once for all women at admission. If the woman was eligible and willing to participate, then the form prompted the data collector to obtain contact information to schedule an interview.

#### Community survey form

**Fig. 5:**
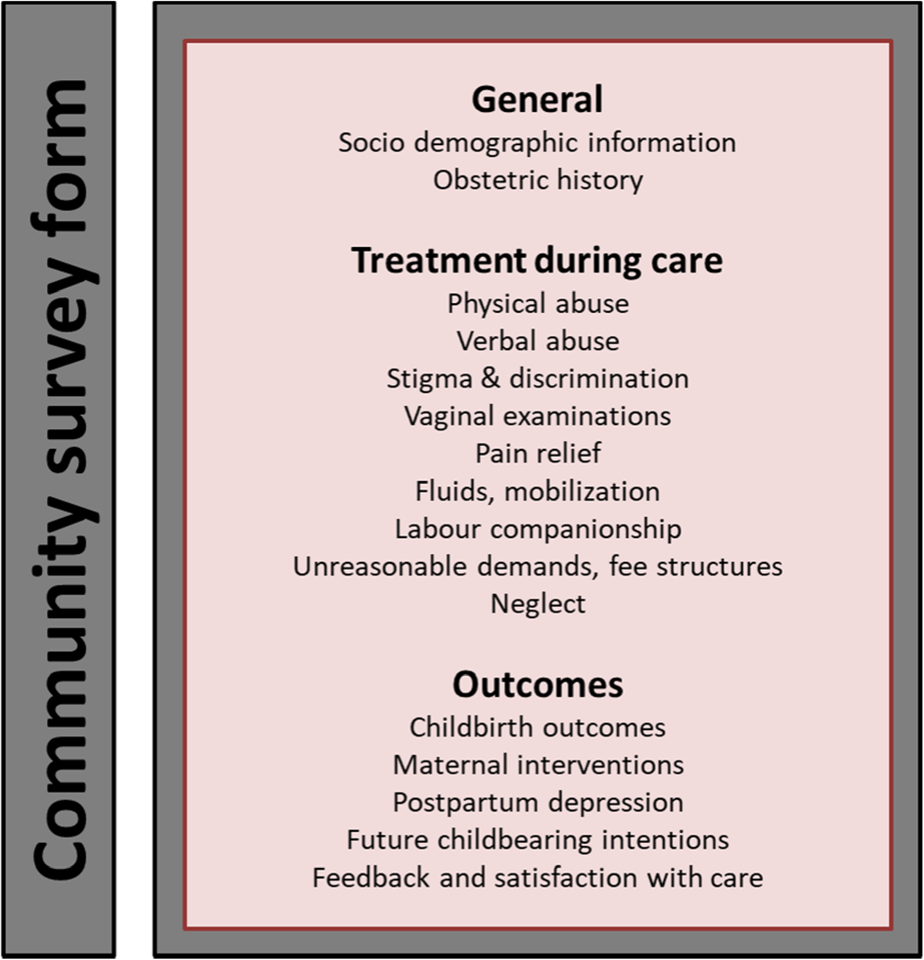
Visual depiction of the structure of the community survey tool

The second form to complete was the community survey form. Figure 5 visually depicts the structure of the community survey form. This form was completed during the follow-up survey, and was completed once for all women. This form captured sociodemographic information, obstetric history, birth experiences (including mistreatment, vaginal examinations, companionship, and pain relief), childbirth outcomes, interventions, postpartum depression, future childbearing intentions and satisfaction with care.

#### Implementation of community survey tool

Overall, 3806 women were screened for the community survey, and 3417 women (89.8%) were eligible to participate. Of the eligible participants, a total of 2672 women (78.2%) completed the community survey: 561 from Nigeria, 644 from Guinea, 836 from Ghana, and 631 from Myanmar. Women who were eligible for participation but did not complete the community survey (745 women, 21.8%) were excluded because they did not give consent (100 women, 13.4%), were unable to be reached by phone (404 women, 54.2%), moved or address was not found (135 women, 18.1%), were referred from the study hospital (9 women, 1.2%), were not contacted because sample size was reached (94 women, 12.6%), or were screened prior to the start of data collection (3 women, 0.4%).

### Additional study documents

Several other study documents were used to manage the recruitment and data collection process: (1) screening log; (2) data submission log; and (3) data collection discrepancy report.

#### Screening log

A screening log was used at each study site to track potential study participants assessed for recruitment in both the labor observation and community survey. Additional file 3: Screening log for the labor observation shows an example of the screening log for the labor observation, and Additional file 4: Screening log for the community survey shows an example of the screening log for the community survey. Each woman arriving at the study facility for childbirth was sequentially assigned a participant number, and information was recorded about her initials, hospital/medical record number, and pre-screening eligibility. The participant number became the unique identifier for each study participant, and was used to anonymize the identity of participants.

#### Data submission log

Each data collector tracked the forms that they submitted on the tablet using a paper-based data submission log (Additional file 5: Data submission log for the labor observation, Additional file 6: Data submission log for the community survey). This log was shared on a weekly basis with the data management team, to ensure that all forms completed and submitted via the tablet were received in the central database. This log helped identify any instances where forms were submitted but not received. This was usually due to a poor 3G connection, where the forms would save locally on the tablet awaiting upload. The data management team would then prompt data collectors to synchronize data using a stronger connection or WiFi.

#### Data collection discrepancy report

In case of any errors on forms that had already been submitted, a data collection discrepancy report was submitted by the research assistant to identify the error and suggest a corrected value (Additional file 7: Data collection discrepancy report). Any discrepancies identified were submitted to the central data management team, and corrections were managed in the central database.

### Linking participant data in the labor observation and community survey

Some women were eligible for participation in both the labor observation and community survey, and participation in one component did not exclude participation from the other component. For this group, collecting the unique hospital/medical record number on the screening form and data collection forms allowed for the labor observation and community survey records to be linked. Linked data allows for a comparison of the birth experience from two perspectives: the woman-reported community survey and the independent observation of labor. Of the 2016 women who participated in the labor observations across the three countries, 1536 women (76.2%) also participated in the community survey and have linked data. This includes 779 women in Ghana (84.1%), 425 women in Guinea (62.3%), 332 women in Nigeria (81.4%).

## Discussion

### Key findings and future analyses

The mistreatment of women during childbirth is globally recognized as a serious issue threatening maternal health and well-being, but there is no consensus on how to best measure this phenomenon to monitor and track progress. This paper presents the development of two tools (labor observation and community survey) to measure the mistreatment of women during childbirth. A mixed-methods, iterative approach was used to develop these tools, which are now available for use. We encourage other researchers to use these tools to measure mistreatment during childbirth occurring in their contexts, and ultimately to improve women’s health and birth experiences globally.

Analysis for this study is ongoing across several domains. First, multi-country epidemiological analyses are underway to assess women’s and newborn’s experiences of mistreatment and health and well-being outcomes, based on both the labor observation and community survey data. Second, psychometric analyses are being conducted to validate two scales (labor observation and community survey) to measure the mistreatment of women during childbirth based on the multi-country data. Once these analyses are completed, we expect to propose two validated scales for measuring the mistreatment of women during childbirth through labor observation and community surveys, and have a better understanding of the magnitude and types of mistreatment that women and newborns experience during childbirth in Ghana, Guinea, Myanmar and Nigeria.

Researchers and program managers may find it useful to embed (or adapt) these tools into quality improvement programs, though further research would be needed to assess their use in this context. For example, they could explore how the mistreatment survey questions may be integrated into other quality improvement measurement tools, such as facility-exit interviews. Likewise, it may be possible for aspects of the labor observation tool to be integrated into facility-based assessments, such as a routine visit to and observation of the labor ward.

### Limitations and strengths

A key strength of this study is that it was conducted in twelve health facilities and community catchment areas across four countries, and involved a multi-disciplinary team of researchers with backgrounds in social sciences, midwifery, obstetrics, and public health. We used a rigorous approach to develop the tools, including a mixed-methods systematic review, primary qualitative research, and systematic mapping of existing tools.

The research team decided to use tablet-based data collection for this study, in part to ensure confidentiality of responses during the labor observations. Tablet-based data collection had strengths and limitations, both from a data collection and data management perspective. Tablets made data collection faster and more user-friendly. The immediate data upload to the central database improved efficiencies in communication between data collectors and data managers. The use of tablets ensured that the tools and participant responses were confidential, which was particularly important for labor observations in clinical environments. Skip patterns and data validation checks built into the tablet forms improved data quality.

However, the OpenClinica software was sometimes cumbersome for the data collectors, as it was not possible to prompt the questions (e.g.: to view part or all of the questions at a glance), which impacted the time spent to complete the forms in cases where new information emerged. After submitting the forms to the server, data collectors could not retrieve the data, so any revisions or changes had to be processed through the central data management unit through submission of data discrepancy reports. This process ensured consistency and transparency to any revisions to the data, but was an additional burden for the data collectors. Due to the specifics of the incident report form structure, it was sometimes challenging for data collectors to capture complex incidents (e.g.: multiple forms of mistreatment happening concurrently by multiple people). To ensure a reliable and complete report of a complex incident, data collectors wrote down the details of the incident and completed the incident report once the situation resolved. Finally, battery life was variable on the tablets; because unreliable electricity sources in some study settings, data collectors were given power banks.

Measuring the mistreatment of women during childbirth is a complex task. We chose to prioritize the development of direct observations of labor and a community-based follow up survey, over facility exit interviews with women. Although facility exit interviews may be logistically more feasible to implement routinely, women may underreport mistreatment while in a facility setting (reporting and social desirability bias) [24, 27]. In this study, labor observations were conducted continuously (24 hours a day, seven days a week, with one observer per woman). While resource-intensive, childbirth is an unpredictable event that can occur at any time of day. Less than 24-hour coverage could contribute to selection and truncation bias; for example, women who arrived at the hospital outside of observation times (e.g.: at night) would be excluded from participating in the study. These women may have different characteristics and experiences compared to women giving birth during the day. Similarly, if a woman recruited in the study did not give birth during the period of observation, then her observation would be stopped prematurely, posing a threat of truncation bias (e.g.: incomplete data), as well as an inefficient use of study resources (e.g.: need to oversample for the labor observations due to the expectation of incomplete observations). In this study, a structured handover between data collectors was used, in case the period of observation for a woman overlapped more than one data collector’s shift. Furthermore, the research team sought insight from other researchers who have conducted labor observations and debated the most appropriate characteristics for data collectors facilitating the labor observations. Due to concerns that data collectors with a clinical background (e.g. retired or student midwives, nurses or doctors) may have normalized behaviors that could be categorized as mistreatment, we decided to have data collectors with public health or social work backgrounds. It is possible that this may have impacted their assessments of more clinical aspects of the labor observations, such as vaginal examinations.

### Research and implementation priorities

More research is needed to further refine these tools and optimize measurement of mistreatment during childbirth in facilities, including how to integrate into routine audit and feedback. We acknowledge that direct, one-to-one observations of labor and community-based follow up may be difficult to implement in routine quality improvement. However, much can be learned, adapted, and implemented from these approaches and tools. For example, elements of the labor observation tool may be integrated into routine monitoring visits or service availability and readiness (SARA) assessments, either at the level of the facility or at the level of an individual woman. Likewise, specific modules from the community survey tool may be integrated into population-based surveys or other community-based follow up of postpartum women. Scale development and prevalence analyses are currently ongoing for the labor observations and community surveys; as such, further refinements to both tools is expected.

## Conclusions

The transformative agenda of the SDGs provides a global landscape to address health and gender inequalities, and improve healthcare experiences. Eliminating all forms of the mistreatment of women during childbirth in facilities is an important component of efforts to transform maternity services globally to be centered on the needs of women and their families. To achieve this, measurement tools are required to understand the magnitude and burden of mistreatment across contexts and to reliably measure progress and identify areas where interventions and policies are needed. We used a systematic, mixed-methods approach to develop two tools (labor observation and community survey) to measure the mistreatment of women during childbirth in four countries, and have made these tools openly available in the public domain. We encourage other researchers and program implementers to implement these tools in their contexts when they are interested in measuring the magnitude of mistreatment during childbirth. It is our expectation that these tools will continue to evolve as further studies are conducted. By measuring the mistreatment of women during childbirth, we will be able to design and implement programs and policies to transform maternity services on a global scale.

## Additional files

Additional file 1: Labor observation tool. This file includes the tablet-based labor observation tool used for data collection. (PDF 282 kb)

Additional file 2: Community survey tool. This file includes the tablet-based community survey tool used for data collection. (PDF 376 kb)

Additional file 3: Screening log for the labor observations. This is an example of the participant screening log used for the labor observation. (DOCX 96 kb)

Additional file 4: Screening log for the community survey. This is an example of the participant screening log used for the community survey. (DOCX 83 kb)

Additional file 5: Data submission log for the labor observation. This is an example of the data submission log used to record tablet-based form submission for the labor observation. (DOCX 74 kb)

Additional file 6: Data submission log for the community survey. This is an example of the data submission log used to record tablet-based form submission for the community survey. (DOCX 65 kb)

Additional file 7: Data collection discrepancy report. This is an example of the data collection discrepancy report used by the data collectors to identify and suggest corrected values for any errors on forms that were already submitted. (DOCX 71 kb)

## Abbreviations

LMIC: Low and middle-income countries
SARA: Service availability and readiness
WHO: World Health Organization
