# Supplementary material for: Methodological development of tools to measure how women are treated during facility-based childbirth in four countries: labor observation and community survey
Source: BMC Med Res Methodol. 2018 Nov 15;18:132. doi: 10.1186/s12874-018-0603-x (PMC6238369; doi:10.1186/s12874-018-0603-x)
Supplement: Supplementary file 1 — Labor observation tool. This file includes the tablet-based labor observation tool used for data collection. (PDF 282 kb) [file 12874_2018_603_MOESM1_ESM.pdf]

Appendix 1. Labor observation tool

LOT - Admission

GENERAL PARTICIPANT

Country code:

GHA

Centre number:

- ◯ 3090 - Korle-Bu Teaching Hospital
- ◯ 3091 - La General Hospital
- ◯ 3092 - Greater Accra Regional (Ridge) Hospital

Medical Record Number:

Participant Number:
Note: From Screening Log

Confirm Participant Number:

SCREENING

1. a) Is the woman being admitted to this hospital for childbirth?

- ◯ No
- ◯ Yes

b) Is the woman being transferred to another hospital immediately, or taken straight to the theatre (for caesarean section)?

- ◯ No
- ◯ Yes

c) Is the woman being admitted for abortion or abortion-related complications?

- ◯ No
- ◯ Yes

d) Is the woman's cervical dilatation 6 cm or less?

- ◯ No
- ◯ Yes

e) Is the woman >= 15 years old?

- ◯ No
- ◯ Yes

f) Is the woman a member of staff or a first degree relation to staff of the facility?

- ◯ No
- ◯ Yes

Provide written information on the study to the woman.

g) Is the woman willing and able to participate?

- ◯ No
- ◯ Yes

Women are **eligible** to participate if they:

Qa= Yes [Are admitted to this hospital for childbirth] AND

Qb= No [Are not being transferred to another hospital immediately or taken straight to theatre for caesarean section] AND

Qc= No [are not admitted for abortion or abortion-related complications] AND

Qd= Yes [Have cervical dilatation <= 6cm (are in early established labour)] AND

Qe= Yes [Are >= 15 years old] AND

Qf= No [Are not related to an employee of the facility] AND

Qg= Yes [Are willing and able to participate]

h) Is the woman eligible?

- ◯ Not Eligible
- ◯ Eligible

If Not eligible, sign and date and stop Labour observation questionnaire here and submit.

If Eligible, offer consent form and provide time to the woman to review consent form.

i) Has the woman provided written consent to participate?

- ◯ No
- ◯ Yes

If No, Stop the Labour observation questionnaire here and submit.

If yes, the woman is now recruited to the study. Complete Admission questionnaire

GENERAL

1. a) Date observation began:

yyyy-mm-dd

b) Time observation began:
(00:00-23:59)

c) Date of admission to hospital:

yyyy-mm-dd

d) Date of admission to labor ward:

yyyy-mm-dd

e) Time of admission to labor ward:
(00:00-23:59)

GENERAL CONTINUED

f) Age:
(years)

____

g) Marital Status:

- ◯ Single / separated / divorced / widowed
- ◯ Married / cohabiting
- ◯ Non-cohabiting partner
- ◯ Other
- ◯ Unknown / don't know

h) Highest level of education attained (from primary to postgraduate):

- ◯ No education
- ◯ Pre-primary education
- ◯ Incomplete primary education
- ◯ Complete primary education
- ◯ Incomplete secondary education
- ◯ Complete secondary education
- ◯ Incomplete post-secondary/ tertiary education
- ◯ Complete post-secondary/tertiary education
- ◯ Other
- ◯ Vocational training
- ◯ Unknown

GENERAL CONTINUED

i) Number of pregnancies (including current pregnancy):
99 if unknown

____

j) Number of previous births (excluding current delivery):
99 if unknown

____

k) Number of previous abortions:
99 if unknown

____

l) Number of previous miscarriages:
99 if unknown

____

m) Number of previous caesarean sections:
99 if unknown

____

n) Is the woman HIV positive:

- ○ No
- ○ Yes
- ○ Don't know

Comment:

____

Each form should be signed, dated and timed by one observer:

Observer code:

____

Date completed:

yyyy-mm-dd

____

Time completed:
(00:00-23:59)

____

END OBSERVATION

Commence observation, complete next section of the observation.

LOT - Incident Report

Country code:

GHA

Centre number:

- ◯ 3090 - Korle-Bu Teaching Hospital
- ◯ 3091 - La General Hospital
- ◯ 3092 - Greater Accra Regional (Ridge) Hospital

Medical Record Number:

Participant Number:
Note: From Screening Log

Confirm Participant Number:

This form should be completed and submitted for every incident that occurs, at the time that it occurs. For example, if a woman is pinched at 12:15 and pinched again at 12:30, these are considered two separate events and two forms should be submitted outlining the events. If a woman is pinched and mocked at the same time at 12:15, then this is considered one event and one form should be submitted outlining this event.

- ☐ Physical Abuse
- ☐ Verbal abuse
- ☐ Stigma and discrimination
- ☐ Vaginal examination

PHYSICAL ABUSE

During the observation period the woman was:

- ☐ Pinched
- ☐ Kicked
- ☐ Slapped
- ☐ Punched
- ☐ Hit with an instrument
- ☐ Gagged (something put across or in her mouth to prevent her from speaking or making noise)
- ☐ Physically tied to the bed (eg: with linen or ropes)
- ☐ Held down to the bed forcefully
- ☐ Given forceful downward pressure directly onto women's abdomen (i.e. fundal pressure)
- ☐ A subject to other use of physical force

2. a) 1. During the observation period, was the woman pinched?

- ◯ No
- ◯ Yes

*** 2. When was the woman pinched?**

- ◯ Intrapartum
- ◯ Postpartum
- ◯ Unknown

*** 3. Time occurred:**
(00:00-23:59)

____

*** 4. Who did it?**

- ◯ Doctor
- ◯ Midwife or Nurse
- ◯ Trainees (Medical student, Nursing or midwifery student)
- ◯ Non-clinical staff (cleaner, administrator, other)
- ◯ Family member or companion of the woman
- ◯ Unknown

If more than one person did this, please press + and complete the question for the other person/people who did this.

2. b) 1. During the observation period, was the woman kicked?

- ◯ No
- ◯ Yes

*** 2. When was the woman kicked?**

- ◯ Intrapartum
- ◯ Postpartum
- ◯ Unknown

*** 3. Time occurred:**
(00:00-23:59)

____

*** 4. Who did it?**

- ◯ Doctor
- ◯ Midwife or Nurse
- ◯ Trainees (Medical student, Nursing or midwifery student)
- ◯ Non-clinical staff (cleaner, administrator, other)
- ◯ Family member or companion of the woman
- ◯ Unknown

If more than one person did this, please press + and complete the question for the other person/people who did this.

2. c) 1. During the observation period, was the woman slapped?

- ◯ No
- ◯ Yes

*** 2. When was the woman slapped?**

- ◯ Intrapartum
- ◯ Postpartum
- ◯ Unknown

*** 3. Time occurred:**
(00:00-23:59)

____

*** 4. Who did it?**

- ◯ Doctor
- ◯ Midwife or Nurse
- ◯ Trainees (Medical student, Nursing or midwifery student)
- ◯ Non-clinical staff (cleaner, administrator, other)
- ◯ Family member or companion of the woman
- ◯ Unknown

If more than one person did this, please press + and complete the question for the other person/people who did this.

2. d) 1. During the observation period, was the woman punched?

- ◯ No
- ◯ Yes

*** 2. When was the woman punched?**

- ◯ Intrapartum
- ◯ Postpartum
- ◯ Unknown

*** 3. Time occurred:**
(00:00-23:59)

____

*** 4. Who did it?**

- ◯ Doctor
- ◯ Midwife or Nurse
- ◯ Trainees (Medical student, Nursing or midwifery student)
- ◯ Non-clinical staff (cleaner, administrator, other)
- ◯ Family member or companion of the woman
- ◯ Unknown

If more than one person did this, please press + and complete the question for the other person/people who did this.

2. e) 1. During the observation period, was the woman hit with an instrument?

- No
- Yes

*** 2. When was the woman hit witn an instrument?**

- Intrapartum
- Postpartum
- Unknown

*** 3. Time occurred:**
(00:00-23:59)

____

*** 4. Who did it?**

- Doctor
- Midwife or Nurse
- Trainees (Medical student, Nursing or midwifery student)
- Non-clinical staff (cleaner, administrator, other)
- Family member or companion of the woman
- Unknown

If more than one person did this, please press + and complete the question for the other person/people who did this.

2. f) 1. During the observation period, was the woman gagged (something put across or in her mouth to prevent her from speaking or making noise)?

- ◯ No
- ◯ Yes

*** 2. When was the woman gagged?**

- ◯ Intrapartum
- ◯ Postpartum
- ◯ Unknown

*** 3. Time occurred:**
(00:00-23:59)

____

*** 4. Who did it?**

- ◯ Doctor
- ◯ Midwife or Nurse
- ◯ Trainees (Medical student, Nursing or midwifery student)
- ◯ Non-clinical staff (cleaner, administrator, other)
- ◯ Family member or companion of the woman
- ◯ Unknown

If more than one person did this, please press + and complete the question for the other person/people who did this.

2. g) 1. During the observation period, was the woman physically tied to the bed (eg: with linen or ropes)?

- No
- Yes

*** 2. When was the woman physically tied to the bed?**

- Intrapartum
- Postpartum
- Unknown

*** 3. Time occurred:**
(00:00-23:59)

____

*** 3. Who did it?**

- Doctor
- Midwife or Nurse
- Trainees (Medical student, Nursing or midwifery student)
- Non-clinical staff (cleaner, administrator, other)
- Family member or companion of the woman
- Unknown

If more than one person did this, please press + and complete the question for the other person/people who did this.

2. h) 1. During the observation period, was the woman held down to the bed forcefully?

- ◯ No
- ◯ Yes

*** 2. When was the woman held down to the bed forcefully?**

- ◯ Intrapartum
- ◯ Postpartum
- ◯ Unknown

*** 3. Time occurred:**
(00:00-23:59)

____

*** 4. Who did it?**

- ◯ Doctor
- ◯ Midwife or Nurse
- ◯ Trainees (Medical student, Nursing or midwifery student)
- ◯ Non-clinical staff (cleaner, administrator, other)
- ◯ Family member or companion of the woman
- ◯ Unknown

If more than one person did this, please press + and complete the question for the other person/people who did this.

2. i) 1. During the observation period, was the woman given forceful downward pressure directly onto women's abdomen (i.e. fundal pressure)?

- ◯ No
- ◯ Yes

*** 2. When was the woman given forceful downward pressure directly onto women's abdomen (i.e. fundal pressure)?**

- ◯ Intrapartum
- ◯ Postpartum
- ◯ Unknown

*** 3. Time occurred:**

(00:00-23:59)

____

*** 4. Who did it?**

- ◯ Doctor
- ◯ Midwife or Nurse
- ◯ Trainees (Medical student, Nursing or midwifery student)
- ◯ Non-clinical staff (cleaner, administrator, other)
- ◯ Family member or companion of the woman
- ◯ Unknown

If more than one person did this, please press + and complete the question for the other person/people who did this.

2. j) 1. Other use of physical force against the mother:

- ◯ No
- ◯ Yes

* **Please specify other use of physical force against the mother:**

____

* **2. When did it occur?**

- ◯ Intrapartum
- ◯ Postpartum
- ◯ Unknown

* **3. Time occurred:**
(00:00-23:59)

____

* **4. Who did it?**

- ◯ Doctor
- ◯ Midwife or Nurse
- ◯ Trainees (Medical student, Nursing or midwifery student)
- ◯ Non-clinical staff (cleaner, administrator, other)
- ◯ Family member or companion of the woman
- ◯ Unknown

If more than one person did this, please press + and complete the question for the other person/people who did this.

VERBAL ABUSE

During the observation period, the woman:

- ☐ Was shouted or screamed at
- ☐ Was insulted
- ☐ Was scolded
- ☐ Was mocked
- ☐ Received negative comments about her physical appearance (including her weight, genitalia, cleanliness or other aspects of a woman's body)
- ☐ Received negative comments about the baby's physical appearance (including his/her appearance, sex, or other aspects of the baby)
- ☐ Received comments about her sexual activity
- ☐ Was threatened with the use of a medical procedure (such as episiotomy, caesarean section or other procedure)
- ☐ Was threatened with physical violence
- ☐ Was threatened that if she does not comply, her or her baby will have a poor outcome
- ☐ Was threatened with withholding care from her or her baby
- ☐ Was blamed for her or her baby's poor health or outcomes
- ☐ Was hissed at
- ☐ Received other form of verbal abuse

3. a) 1. During the observation period, was the woman shouted or screamed at?

- No
- Yes

*** 2. When was the woman shouted or screamed at?**

- Intrapartum
- Postpartum
- Unknown

*** 3. Time occurred:**
(00:00-23:59)

____

*** 4. Who did it?**

- Doctor
- Midwife or Nurse
- Trainees (Medical student, Nursing or midwifery student)
- Non-clinical staff (cleaner, administrator, other)
- Family member or companion of the woman
- Unknown

If more than one person did this, please press + and complete the question for the other person/people who did this.

3. b) 1. During the observation period, was the woman insulted?

- No
- Yes

*** 2. When was the woman insulted?**

- Intrapartum
- Postpartum
- Unknown

*** 3. Time occurred:**

(00:00-23:59)

____

*** 4. Who did it?**

- Doctor
- Midwife or Nurse
- Trainees (Medical student, Nursing or midwifery student)
- Non-clinical staff (cleaner, administrator, other)
- Family member or companion of the woman
- Unknown

If more than one person did this, please press + and complete the question for the other person/people who did this.

3. c) 1. During the observation period, was the woman scolded?

- No
- Yes

*** 2. When was the woman scolded?**

- Intrapartum
- Postpartum
- Unknown

*** 3. Time occurred:**
(00:00-23:59)

____

*** 4. Who did it?**

- Doctor
- Midwife or Nurse
- Trainees (Medical student, Nursing or midwifery student)
- Non-clinical staff (cleaner, administrator, other)
- Family member or companion of the woman
- Unknown

If more than one person did this, please press + and complete the question for the other person/people who did this.

3. d) 1. During the observation period, was the woman mocked?

- ◯ No
- ◯ Yes

*** 2. When was the woman mocked?**

- ◯ Intrapartum
- ◯ Postpartum
- ◯ Unknown

*** 3. Time occurred:**
(00:00-23:59)

____

*** 4. Who did it?**

- ◯ Doctor
- ◯ Midwife or Nurse
- ◯ Trainees (Medical student, Nursing or midwifery student)
- ◯ Non-clinical staff (cleaner, administrator, other)
- ◯ Family member or companion of the woman
- ◯ Unknown

If more than one person did this, please press + and complete the question for the other person/people who did this.

3. e) 1. During the observation period, did the woman receive negative comments about her physical appearance (including her weight, genitalia, cleanliness or other aspects of a woman's body)?

- ◯ No
- ◯ Yes

* **2. When did the woman receive negative comments about her physical appearance (including her weight, genitalia, cleanliness or other aspects of a woman's body)?**

- ◯ Intrapartum
- ◯ Postpartum
- ◯ Unknown

* **3. Time occurred:**

(00:00-23:59)

____

* **4. Who did it?**

- ◯ Doctor
- ◯ Midwife or Nurse
- ◯ Trainees (Medical student, Nursing or midwifery student)
- ◯ Non-clinical staff (cleaner, administrator, other)
- ◯ Family member or companion of the woman
- ◯ Unknown

If more than one person did this, please press + and complete the question for the other person/people who did this.

3. f) 1. During the observation period, did the woman receive negative comments about the baby's physical appearance (including his/her appearance, sex, or other aspects of the baby)?

- ◯ No
- ◯ Yes

*** 2. When did the woman receive negative comments about the baby's physical appearance (including his/her appearance, sex, or other aspects of the baby)?**

- ◯ Intrapartum
- ◯ Postpartum
- ◯ Unknown

*** 3. Time occurred:**

(00:00-23:59)

____

*** 4. Who did it?**

- ◯ Doctor
- ◯ Midwife or Nurse
- ◯ Trainees (Medical student, Nursing or midwifery student)
- ◯ Non-clinical staff (cleaner, administrator, other)
- ◯ Family member or companion of the woman
- ◯ Unknown

If more than one person did this, please press + and complete the question for the other person/people who did this.

3. g) 1. During the observation period, did the woman receive comments about her sexual activity?

- No
- Yes

*** 2. When did the woman receive comments about her sexual activity?**

- Intrapartum
- Postpartum
- Unknown

*** 3. Time occurred:**
(00:00-23:59)

____

*** 4. Who did it?**

- Doctor
- Midwife or Nurse
- Trainees (Medical student, Nursing or midwifery student)
- Non-clinical staff (cleaner, administrator, other)
- Family member or companion of the woman
- Unknown

If more than one person did this, please press + and complete the question for the other person/people who did this.

3. h) 1. During the observation period, was the woman threatened with use of a medical procedure (such as episiotomy, caesarean section or other procedure)?

- No
- Yes

*** 2. When was the woman threatened with use of a medical procedure (such as episiotomy, caesarean section or other procedure)?**

- Intrapartum
- Postpartum
- Unknown

*** 3. Time occurred:**

(00:00-23:59)

____

*** 4. Who did it?**

- Doctor
- Midwife or Nurse
- Trainees (Medical student, Nursing or midwifery student)
- Non-clinical staff (cleaner, administrator, other)
- Family member or companion of the woman
- Unknown

If more than one person did this, please press + and complete the question for the other person/people who did this.

3. i) 1. During the observation period, was the woman threatened with physical violence?

- No
- Yes

*** 2. When was the woman threatened with physical violence?**

- Intrapartum
- Postpartum
- Unknown

*** 3. Time occurred:**
(00:00-23:59)

____

*** 4. Who did it?**

- Doctor
- Midwife or Nurse
- Trainees (Medical student, Nursing or midwifery student)
- Non-clinical staff (cleaner, administrator, other)
- Family member or companion of the woman
- Unknown

If more than one person did this, please press + and complete the question for the other person/people who did this.

3. j) 1. During the observation period, was the woman threatened that if she does not comply, her or her baby will have a poor outcome?

- ◯ No
- ◯ Yes

*** 2. When was the woman threatened that if she does not comply, her or her baby will have a poor outcome?**

- ◯ Intrapartum
- ◯ Postpartum
- ◯ Unknown

*** 3. Time occurred:**
(00:00-23:59)

____

*** 4. Who did it?**

- ◯ Doctor
- ◯ Midwife or Nurse
- ◯ Trainees (Medical student, Nursing or midwifery student)
- ◯ Non-clinical staff (cleaner, administrator, other)
- ◯ Family member or companion of the woman
- ◯ Unknown

If more than one person did this, please press + and complete the question for the other person/people who did this.

3. k) 1. During the observation period, was the woman threatened with withholding care from her or her baby?

- ◯ No
- ◯ Yes

*** 2. When was the woman threatened with withholding care from her or her baby?**

- ◯ Intrapartum
- ◯ Postpartum
- ◯ Unknown

*** 3. Time occurred:**
(00:00-23:59)

____

*** 4. Who did it?**

- ◯ Doctor
- ◯ Midwife or Nurse
- ◯ Trainees (Medical student, Nursing or midwifery student)
- ◯ Non-clinical staff (cleaner, administrator, other)
- ◯ Family member or companion of the woman
- ◯ Unknown

If more than one person did this, please press + and complete the question for the other person/people who did this.

3. l) 1. During the observation period, was the woman blamed for her or her baby's poor health or outcomes?

- ◯ No
- ◯ Yes

*** 2. When was the woman blamed for her or her baby's poor health or outcomes?**

- ◯ Intrapartum
- ◯ Postpartum
- ◯ Unknown

*** 3. Time occurred:**
(00:00-23:59)

____

*** 4. Who did it?**

- ◯ Doctor
- ◯ Midwife or Nurse
- ◯ Trainees (Medical student, Nursing or midwifery student)
- ◯ Non-clinical staff (cleaner, administrator, other)
- ◯ Family member or companion of the woman
- ◯ Unknown

If more than one person did this, please press + and complete the question for the other person/people who did this.

3. m) 1. During the observation period, was the woman hissed at?

- ◯ No
- ◯ Yes

*** 2. When was the woman was hissed at?**

- ◯ Intrapartum
- ◯ Postpartum
- ◯ Unknown

*** 3. Time occurred:**
(00:00-23:59)

____

*** 4. Who did it?**

- ◯ Doctor
- ◯ Midwife or Nurse
- ◯ Trainees (Medical student, Nursing or midwifery student)
- ◯ Non-clinical staff (cleaner, administrator, other)
- ◯ Family member or companion of the woman
- ◯ Unknown

If more than one person did this, please press + and complete the question for the other person/people who did this.

3. n) 1. Did the woman experience other forms of verbal abuse?

- ◯ No
- ◯ Yes

* **Please specify other forms of verbal abuse:**

____

* **2. When did it occur?**

- ◯ Intrapartum
- ◯ Postpartum
- ◯ Unknown

* **3. Time occurred:**
(00:00-23:59)

____

* **4. Who did it?**

- ◯ Doctor
- ◯ Midwife or Nurse
- ◯ Trainees (Medical student, Nursing or midwifery student)
- ◯ Non-clinical staff (cleaner, administrator, other)
- ◯ Family member or companion of the woman
- ◯ Unknown

If more than one person did this, please press + and complete the question for the other person/people who did this.

STIGMA AND DISCRIMINATION

During the observation period, the woman:

- ☐ Received negative comments about her ethnicity or race
- ☐ Received negative comments about her religion
- ☐ Received negative comments about her age
- ☐ Received negative comments about her marital status
- ☐ Received negative comments about her education or literacy level
- ☐ Received negative comments about her lower economic circumstances (eg: poverty)
- ☐ Received negative comments about her higher economic circumstances (e.g: wealth)
- ☐ Received other instances of stigma or discrimination

*** 4. a) 1. During the observation period, did the woman receive negative comments about her ethnicity or race?**

- ◯ No
- ◯ Yes

*** 2. When did the woman receive negative comments about her ethnicity or race?**

- ◯ Intrapartum
- ◯ Postpartum
- ◯ Unknown

*** 3. Time occurred:**
(00:00-23:59)

____

*** 4. Who did it?**

- ◯ Doctor
- ◯ Midwife or Nurse
- ◯ Trainees (Medical student, Nursing or midwifery student)
- ◯ Non-clinical staff (cleaner, administrator, other)
- ◯ Family member or companion of the woman
- ◯ Unknown

If more than one person did this, please press + and complete the question for the other person/people who did this.

*** 4. b) 1. During the observation period, did the woman receive negative comments about her religion?**

- No
- Yes

*** 2. When did the woman receive negative comments about her religion?**

- Intrapartum
- Postpartum
- Unknown

*** 3. Time occurred:**
(00:00-23:59)

____

*** 4. Who did it?**

- Doctor
- Midwife or Nurse
- Trainees (Medical student, Nursing or midwifery student)
- Non-clinical staff (cleaner, administrator, other)
- Family member or companion of the woman
- Unknown

If more than one person did this, please press + and complete the question for the other person/people who did this.

*** 4. c) 1. During the observation period, did the woman receive negative comments about her age?**

- ◯ No
- ◯ Yes

*** 2. When did the woman receive negative comments about her age?**

- ◯ Intrapartum
- ◯ Postpartum
- ◯ Unknown

*** 3. Time occurred:**
(00:00-23:59)

____

*** 4. Who did it?**

- ◯ Doctor
- ◯ Midwife or Nurse
- ◯ Trainees (Medical student, Nursing or midwifery student)
- ◯ Non-clinical staff (cleaner, administrator, other)
- ◯ Family member or companion of the woman
- ◯ Unknown

If more than one person did this, please press + and complete the question for the other person/people who did this.

*** 4. d) 1. During the observation period, did the woman receive negative comments about her marital status?**

- ○ No
- ○ Yes

*** 2. When did the woman receive negative comments about her marital status?**

- ○ Intrapartum
- ○ Postpartum
- ○ Unknown

*** 3. Time occurred:**
(00:00-23:59)

____

*** 4. Who did it?**

- ○ Doctor
- ○ Midwife or Nurse
- ○ Trainees (Medical student, Nursing or midwifery student)
- ○ Non-clinical staff (cleaner, administrator, other)
- ○ Family member or companion of the woman
- ○ Unknown

If more than one person did this, please press + and complete the question for the other person/people who did this.

*** 4. e) 1. During the observation period, did the woman receive negative comments about her education or literacy level?**

- No
- Yes

*** 2. When did the woman receive negative comments about her education or literacy level?**

- Intrapartum
- Postpartum
- Unknown

*** 3. Time occurred:**
(00:00-23:59)

*** 4. Who did it?**

- Doctor
- Midwife or Nurse
- Trainees (Medical student, Nursing or midwifery student)
- Non-clinical staff (cleaner, administrator, other)
- Family member or companion of the woman
- Unknown

If more than one person did this, please press + and complete the question for the other person/people who did this.

*** 4. f) 1. During the observation period, did the woman receive negative comments about her lower economic circumstances (e.g. poverty)?**

- ◯ No
- ◯ Yes

*** 2. When did the woman receive negative comments about her lower economic circumstances (e.g. poverty)?**

- ◯ Intrapartum
- ◯ Postpartum
- ◯ Unknown

*** 3. Time occurred:**
(00:00-23:59)

____

*** 4. Who did it?**

- ◯ Doctor
- ◯ Midwife or Nurse
- ◯ Trainees (Medical student, Nursing or midwifery student)
- ◯ Non-clinical staff (cleaner, administrator, other)
- ◯ Family member or companion of the woman
- ◯ Unknown

If more than one person did this, please press + and complete the question for the other person/people who did this.

*** 4. g) 1. During the observation period, did the woman receive negative comments about her higher economic circumstances (e.g. wealth)?**

- ○ No
- ○ Yes

*** 2. When did the woman receive negative comments about her higher economic circumstances (e.g. wealth)?**

- ○ Intrapartum
- ○ Postpartum
- ○ Unknown

*** 3. Time occurred:**

(00:00-23:59)

____

*** 4. Who did it?**

- ○ Doctor
- ○ Midwife or Nurse
- ○ Trainees (Medical student, Nursing or midwifery student)
- ○ Non-clinical staff (cleaner, administrator, other)
- ○ Family member or companion of the woman
- ○ Unknown

If more than one person did this, please press + and complete the question for the other person/people who did this.

*** 4. h) 1. During the observation period, were there other instances of stigma or discrimination against the woman?**

- ◯ No
- ◯ Yes

*** Please specify other instances of stigma or discrimination against the woman:**

____

*** 2. When did it occur?**

- ◯ Intrapartum
- ◯ Postpartum
- ◯ Unknown

*** 3. Time occurred:**
(00:00-23:59)

____

*** 4. Who did it?**

- ◯ Doctor
- ◯ Midwife or Nurse
- ◯ Trainees (Medical student, Nursing or midwifery student)
- ◯ Non-clinical staff (cleaner, administrator, other)
- ◯ Family member or companion of the woman
- ◯ Unknown

If more than one person did this, please press + and complete the question for the other person/people who did this.

VAGINAL EXAMINATION

During the observation period, did the woman receive any vaginal examination?

- No
- Yes
- Unknown

5. a) Before giving a vaginal examination, did the staff inform the woman of why a vaginal examination is needed?

- No
- Yes
- Unknown

5. b) Did the staff member obtain permission of the woman before the vaginal examination?

- No
- Yes
- Unknown

5. c) Did a staff member discuss the woman's private health information in a way that others (non-medical staff, other patients or other patients' family members) could hear?

- No
- Yes
- Unknown

5. d) Did a staff member conduct a vaginal examination in a way that others (patients, visitors, non-medical staff) could see her genitalia?

- No
- Yes
- Unknown

5. e) Did the staff member conduct a vaginal examination in a way that others (patients, visitors, non-medical staff) could see her breasts?

- No
- Yes
- Unknown

Comment:

Each form should be signed, dated and timed by one observer:

Observer code:

____

Date completed:

yyyy-mm-dd

____

Time completed:

(00:00-23:59)

____

LOT - Inpatient care - Childbirth

INPATIENT PARTICIPANT

Country code:

GHA

Centre number:

- ◯ 3090 - Korle-Bu Teaching Hospital
- ◯ 3091 - La General Hospital
- ◯ 3092 - Greater Accra Regional (Ridge) Hospital

Medical Record Number:

Participant Number:
Note: From Screening Log

Confirm Participant Number:

This form should be completed and submitted for all women at the end of the observation period. In case of handing over to another observer while the woman is still in labour, data collectors should discuss which part of the form they have completed to ensure proper handover. The new data collector will then continue completing this form."

During the observation period did any of the following events occur? (select/tick all to complete this section)

- ☐ The woman was offered and/or requested a pain relief.
- ☐ The woman had easy access to water or oral fluids.
- ☐ The woman was encouraged and/or was able to mobilize.
- ☐ The woman was offered to have/had a labour companion.
- ☐ The woman was a subject to unreasonable demands, fee structure and/or neglect.
- ☐ None of the above.

PAIN RELIEF

6. During the observation period, was the woman offered any form of pain relief?

- No
- Yes
- Unknown

7. During the observation period, did the woman request pain relief?

- No
- Yes
- Unknown

a) If Yes, was the woman given pain relief?

- No
- Yes
- Unknown

FLUIDS

8. a) During labour, did the woman have easy access to water or oral fluids?

- No
- Yes
- Unknown

MOBILIZATION

b) Was the woman told she could mobilize during labour?

- No
- Yes
- Unknown

c) Did woman mobilize during labour?

- No
- Yes
- Unknown

COMPANION

9. a) Was the woman offered to have a labour companion during labour and delivery?

- ◯ No
- ◯ Yes
- ◯ Unknown

b) 1. Did the woman have a companion present at any time during her labour and delivery?

- ◯ No
- ◯ Yes
- ◯ Unknown

2. If Yes, Who was the companion?

- ◯ Husband / male partner
- ◯ Family member
- ◯ Friend
- ◯ Doula
- ◯ Traditional birth attendant
- ◯ Other

If Other, specify

____

UNREASONABLE DEMANDS, FEE STRUCTURES AND NEGLECT

10. At any time, was the woman instructed to clean up blood, urine, faeces or amniotic fluid?

- ◯ No
- ◯ Yes
- ◯ Unknown

11. At any time, did staff suggest or ask the woman (or companion) for a bribe, informal payment or gift?

- ◯ No
- ◯ Yes
- ◯ Unknown

12. Did the woman request medical attention from a health worker that was not responded to?

- ◯ No
- ◯ Yes
- ◯ Unknown

Each form should be signed, dated and timed by one observer:

Observer code:

____

Date completed:

yyyy-mm-dd

Time completed:

(00:00-23:59)

This form should be completed after the baby is born. Some questions in this section refer to the period immediately around childbirth, other questions refer to status at the end of the observation period. This form should be finalized and submitted when the observation period is completed (either 2 hours postpartum, discharge from the facility, or transfer from the facility, whichever happens first).

CHILDBIRTH

13. a) Date of delivery:

yyyy-mm-dd

b) Time of delivery:
(00:00-23:59)

c) Final mode of delivery:

- ◯ Unassisted vaginal delivery
- ◯ Assisted vaginal delivery (vacuum or forceps)
- ◯ C/section
- ◯ Laparotomy
- ◯ Assisted breech delivery
- ◯ Destructive operation
- ◯ Unknown

d) 1. Did a perineal tear occur?

- ◯ No tear
- ◯ First degree tear
- ◯ Second degree tear
- ◯ Third or fourth degree tear
- ◯ Unknown

2. If a perineal tear occurred, was a perineal repair or suture performed?

- ◯ No
- ◯ Yes
- ◯ Unknown

3. If a perineal repair or suture was performed, was local anaesthetic used?

- ◯ No
- ◯ Yes

Childbirth

At your most recent childbirth, did you have one baby or two babies (twins)?

- ◯ One baby (single birth)
- ◯ Two babies (twins, multiple birth)

*** e) Baby status at birth:**

- ◯ Infant alive at birth
- ◯ Fresh stillbirth
- ◯ Macerated stillbirth
- ◯ Unknown

*** f) Infant sex:**

- ◯ Male
- ◯ Female
- ◯ Unknown

*** g) Birth weight (grams):**
9999 if Unknown

____

*** h) Apgar score at 5 minutes:**
99 if Unknown

____

Press + if entering more than one baby (twins), or press next if entering one baby (single birth)

CHILDBIRTH CONTINUED

i) 1. Did the woman have a companion present when the baby was born?

- ◯ No
- ◯ Yes

2. Who was the companion?

- ◯ Husband / male partner
- ◯ Family member
- ◯ Friend
- ◯ Doula
- ◯ Traditional birth attendant
- ◯ Other

If Other, specify:

____

CHILDBIRTH CONTINUED

j) 1. Did the woman state a preferred birthing position?

- No
- Yes
- Unknown

2. What was the position?

- Dorsal/supine
- Lithotomy
- On all fours
- Squatting
- Sitting
- Lying on her side
- Other
- Unknown

If Other, specify:

____

k) Was the woman asked her preferred birthing position?

- No
- Yes
- Unknown

l) What position did the woman deliver in?

- Dorsal/supine
- Lithotomy
- On all fours
- Squatting
- Sitting
- Lying on her side
- Other
- Unknown

If Other, specify:

____

CHILDBIRTH CONTINUED

14. a) Who was the primary birth attendant for this woman throughout her labour and delivery?

- ◯ No attendant
- ◯ Obstetrician
- ◯ Resident Doctor
- ◯ Midwife/Nurse
- ◯ Auxiliary Midwife
- ◯ Medical Student
- ◯ Midwifery Student
- ◯ Nursing Student
- ◯ Traditional Birth Attendant
- ◯ Other
- ◯ Unknown / Not Specified

If Other, specify:

____

CHILDBIRTH CONTINUED

b) Was a staff member present when the baby came out?

- ◯ No
- ◯ Yes

c) What staff member present?

1. Obstetrician

- ◯ No
- ◯ Yes

2. Resident Doctor

- ◯ No
- ◯ Yes

3. Midwife/Nurse

- ◯ No
- ◯ Yes

4. Auxiliary Midwife

- ◯ No
- ◯ Yes

5. Medical Student

- ◯ No
- ◯ Yes

6. Midwifery Student

- ◯ No
- ◯ Yes

7. Nursing Student

- ◯ No
- ◯ Yes

8. Unknown / not specified

- ◯ No
- ◯ Yes

9. Other

- ◯ No
- ◯ Yes

If Other, specify:

____

CHILDBIRTH CONTINUED

d) 1. Did the woman's primary birth attendant speak the same language as the woman?

- ◯ No
- ◯ Yes
- ◯ Unknown

2. Was an interpreter used?

- ◯ No
- ◯ Yes
- ◯ Unknown

3. Who was the interpreter?

- ◯ Professional interpreter
- ◯ Staff member
- ◯ Family member
- ◯ Another patient
- ◯ Someone else
- ◯ Unknown

If Someone else, specify:

____

CHILDBIRTH CONTINUED

15. a) Were curtains, partitions, or other measures used to provide privacy for the woman during labour (prior to childbirth)?

- ○ No
- ○ Yes
- ○ Unknown

b) Were curtains, partitions, or other measures used to provide privacy for the woman as the baby was coming out, through the delivery of the placenta?

- ○ No
- ○ Yes
- ○ Unknown

c) Were curtains, partitions, or other measures used to provide privacy for the woman during postpartum period?

- ○ No
- ○ Yes
- ○ Unknown

CHILDBIRTH CONTINUED

d) Did the woman have a bed during labour?

- ○ No
- ○ Yes
- ○ Unknown

e) Did the woman have a bed during childbirth?

- ○ No
- ○ Yes
- ○ Unknown

f) Did the woman have a bed during postpartum period?

- ○ No
- ○ Yes
- ○ Unknown

g) At any time, did the woman have to share a bed with another woman or women?

- ○ No
- ○ Yes
- ○ Unknown

CHILDBIRTH CONTINUED

h) Comment:

____

MATERNAL INTERVENTIONS

During the observation period did the woman experience any of the following procedures? (select/tick all that apply)

- ☐ Caesarean section
- ☐ Episiotomy
- ☐ Hysterectomy
- ☐ Tubal ligation / sterilization
- ☐ Postpartum IUD insertion?
- ☐ None of the above

CAESAREAN SECTION

16. a) 1. Did the woman experience caesarean section?

- No
- Yes
- Unknown

2. Was information about the procedure communicated to the woman, including risks and benefits? (select/tick all that apply)

- No
- Yes
- Unknown

3. Did the woman provide her consent prior to the procedure (or, if the woman was physically unable to provide consent, someone provided consent on her behalf)?

- No
- Yes
- Unknown

4. Time of decision:

(00:00-23:59

____

5. Time of incision:

(00:00-23:60

____

6. What was the primary indication for the Caesarean Section:

- Fetal distress
- Prolonged labour
- Cephalopelvic disproportion
- Obstruction
- Suspected/imminent uterine rupture
- Failed assisted vaginal delivery
- Pre-eclampsia/eclampsia
- Gestational age >= 41 weeks
- Intrapartum vaginal bleeding
- Breech or other malpresentation
- Previous CS
- Failed induction
- Maternal request
- HIV
- Genital Herpes/extensive condyloma
- Previous uterine surgery
- Other complication
- Unknown

EPISIOTOMY

b) 1. Did the woman experience episiotomy?

- No
- Yes
- Unknown

2. Was information about the procedure communicated to the woman, including risks and benefits?

- No
- Yes
- Unknown

3. Did the woman provide her consent prior to the procedure (or. If the woman was physically unable to provide consent, someone provided consent on her behalf)?

- No
- Yes
- Unknown

HYSTERECTOMY

c) 1. Did the woman experience hysterectomy?

- No
- Yes
- Unknown

2. Was information about the procedure communicated to the woman, including risks and benefits?

- No
- Yes
- Unknown

3. Did the woman provide her consent prior to the procedure (or. If the woman was physically unable to provide consent, someone provided consent on her behalf)?

- No
- Yes
- Unknown

TUBAL LIGATION / STERILIZATION

d) 1. Did the woman experience tubal ligation / sterilization?

- No
- Yes
- Unknown

2. Was information about the procedure communicated to the woman, including risks and benefits?

- No
- Yes
- Unknown

3. Did the woman provide her consent prior to the procedure (or. If the woman was physically unable to provide consent, someone provided consent on her behalf)?

- No
- Yes
- Unknown

POSTPARTUM IUD INSERTION

e) 1. Did the woman experience postpartum IUD insertion?

- No
- Yes
- Unknown

2. Was information about the procedure communicated to the woman, including risks and benefits?

- No
- Yes
- Unknown

3. Did the woman provide her consent prior to the procedure (or. If the woman was physically unable to provide consent, someone provided consent on her behalf)?

- No
- Yes
- Unknown

MATERNAL INTERVENTIONS CONTINUED

17. Please specify whether the ***woman*** received any of the following inteventions during the observation period:

a) Induction of labour:

- ◯ No
- ◯ Yes
- ◯ Not applicable
- ◯ Unknown

b) Augmentation of labour:

- ◯ No
- ◯ Yes
- ◯ Not applicable
- ◯ Unknown

c) Perineal shaving:

- ◯ No
- ◯ Yes
- ◯ Not applicable
- ◯ Unknown

d) Enema:

- ◯ No
- ◯ Yes
- ◯ Not applicable
- ◯ Unknown

e) Re-stiching of FGM scar:

- ◯ No
- ◯ Yes
- ◯ Not applicable
- ◯ Unknown

NEWBORN INTERVENTIONS

18. Please specify whether the ***newborn*** received any of the following interventions:

a) Cord clamping performed >= 60 seconds after delivery:

- No
- Yes
- Not applicable
- Unknown

b) Immediate skin-to-skin contact with mother:

- No
- Yes
- Not applicable
- Unknown

c) If baby was transferred to another health facility, was parental consent obtained?

- No
- Yes
- Not applicable
- Unknown

d) Breastfeeding on baby's demand:

- No
- Yes
- Not applicable
- Unknown

e) Routine suctioning of newborn:

- No
- Yes
- Not applicable
- Unknown

f) Refusal to provide postnatal care for mother and baby due to inability to pay:

- No
- Yes
- Not applicable
- Unknown

g) Newborn left unattended:

- No
- Yes
- Not applicable
- Unknown

h) Bathing during observation period:

- No
- Yes
- Not applicable
- Unknown

i) Newborn separated from mother after birth:

- No
- Yes
- Not applicable
- Unknown

j) Slapping the newborn:

- No
- Yes
- Not applicable
- Unknown

k) Holding the newborn upside down:

- No
- Yes
- Not applicable
- Unknown

l) Holding the newborn by the leg:

- No
- Yes
- Not applicable
- Unknown

m) Milking the newborn's chest:

- No
- Yes
- Not applicable
- Unknown

n) Flexing the newborn's legs towards the abdomen:

- ◯ No
- ◯ Yes
- ◯ Not applicable
- ◯ Unknown

o) Rubbing the newborn with alcohol:

- ◯ No
- ◯ Yes
- ◯ Not applicable
- ◯ Unknown

p) Breastfeeding within 30 minutes after birth:

- ◯ No
- ◯ Yes
- ◯ Not applicable
- ◯ Unknown

19. OTHER INTERVENTIONS

a) Admission to maternal Intensive Care Unit:

- ◯ No
- ◯ Yes
- ◯ Unknown

b) During the observation period, was the woman referred to another hospital?

- ◯ No
- ◯ Yes
- ◯ Unknown

c) Woman discharged from hospital before end of obervation period:

- ◯ No
- ◯ Yes
- ◯ Unknown

1. Date of discharge:

yyyy-mm-dd

2. Time of discharge:
(00:00-23:59)

d) Maternal status at end of observation period:

- ◯ Alive
- ◯ Dead
- ◯ Unknown

e) Admission to neonatal intensive care unit or special care baby unit by end of observation:

- ◯ No
- ◯ Yes
- ◯ Unknown

f) Newborn status at end of observation period (2 hours postpartum):

- ◯ Infant alive
- ◯ Infant not alive
- ◯ Unknown

g) Date observation concluded:

yyyy-mm-dd

h) Time observation concluded:
(00:00-23:59)

---

20. Results of observation:

- ◯ Observation completed
- ◯ Observation started, but not completed - participation withdrawn
- ◯ Observation started, but not completed - other

21. Comment:

Each form should be signed, dated & timed by one observer:

Observer code:

Date completed:

yyyy-mm-dd

Time completed:
(00:00-23:59)

Please make sure that all other forms are completed and submitted for this observation. For each woman, the following forms should be completed and submitted:

1. Admission

2. Inpatient care, Childbirth, interventions and discharge

3. Incident reports (as applicable)
