# Supplementary material for: Methodological development of tools to measure how women are treated during facility-based childbirth in four countries: labor observation and community survey
Source: BMC Med Res Methodol. 2018 Nov 15;18:132. doi: 10.1186/s12874-018-0603-x (PMC6238369; doi:10.1186/s12874-018-0603-x)
Supplement: Supplementary file 2 — Community survey tool. This file includes the tablet-based community survey tool used for data collection. (PDF 376 kb) [file 12874_2018_603_MOESM2_ESM.pdf]

Appendix 2. Community survey tool

CST - Screening

Country code:

GHA

Centre number:

- ◯ 3090 - Korle-Bu Teaching Hospital
- ◯ 3091 - La General Hospital
- ◯ 3092 - Greater Accra Regional (Ridge) Hospital

Medical Record Number:

Participant Number:
Note: From Screening Log

Confirm Participant Number:

SCREENING

1. a) Were you admitted to this hospital for childbirth? ***Y'agye wo ato asopiti ha ma awoɔ anaa?***

- ◯ No/Dabi
- ◯ Yes/Aane

b) Are you >= 15 years old? ***Wo mfe a w'adi yɛ edunum anaa edununum ne akyire?***

- ◯ No/Dabi
- ◯ Yes/Aane

c) Are you staff or a first degree relation to a staff member at this facility? ***Wo busuani bi yɛ adwuma asopiti ha?***

- ◯ No/Dabi
- ◯ Yes/Aane

d) Will you live within [defined catchment area] of this facility at 2 to 6 weeks after childbirth? ***Mpɔtamu ha na wote?***

- ◯ No/Dabi
- ◯ Yes/Aane

e) Are you willing and able to participate in an interview 2 to 6 weeks after childbirth? ***Wayɛ krado, na wobetumi sɛ wo be ma yɛn nsɛmisa bi ho mmuae Nnawɔtwe mmienu kosi nnan wɔ wawoɔ akyi?***

- ◯ No/Dabi
- ◯ Yes/Aane

Women are eligible to participate if they:

Q1a = Yes [Admitted to this hospital for childbirth], *AND*

Q1b = Yes [Are >= 15 years old], *AND*

Q1c = No [Are not staff or a first degree relation to a staff member at this facility], *AND*

Q1d = Yes [Live within the defined catchment area of the facility 2 to 6 weeks after childbirth] *AND*

Q1e = Yes [Are willing and able to participate in an interview 2-6 weeks after childbirth].

f) Is the woman eligible? ***ɔwɔ ho kwan?***

- ◯ Not Eligible
- ◯ Eligible

If woman is Not eligible, sign and date and stop Community Survey questionnaire here and submit.

g) If eligible, date of recruitment:

yyyy-mm-dd ____________

Offer consent form and provide time to the woman to review consent form.

If the woman is now recruited to the study, complete the Contact form with her information.

Comment:

Observer code:

Date completed:

yyyy-mm-dd

Time completed:
(00:00-23:59)

CST

Country Code:

GHA

Centre number:

- ◯ 3090 - Korle-Bu Teaching Hospital
- ◯ 3091 - La General Hospital
- ◯ 3092 - Greater Accra Regional (Ridge) Hospital

Medical Record Number:

Participant Number:
Note: From Screening Log

Confirm Participant Number:

General

1. a) Has the woman provided written consent to participate? *baa no de ne nsa ahyɛ nwoma ase sɛ obɛma nsɛmisa no ho mmuae?*

- ○ No/dabi
- ○ Yes/Aane

If No, Stop the Community survey questionnaire here

b) Is the identity of the woman confirmed? *Wa tumi ahu obaa no ahyɛnso de?*

- ○ No/dabi
- ○ Yes/Aane

c) Date of delivery: *Da awo no kɔɔ so*

yyyy-mm-dd

d) Date of interview: *Da a nsemisa no kɔɔ so*

yyyy-mm-dd

e) Time of interview: *Bere a nsemisa no kɔɔ so*

(00:00-23:59)

f) Results of interview: *Nsemisa no ho nsonsoanso*

- ○ Survey completed/Nhwehwɛ mu no baa awieɛ
- ○ Survey started but not completed/ Nhwehwɛmu no hyɛɛ ase nanso amma awieɛ
- ○ Woman unavailable at time of visit/Na ɔbaa no nni hɔ bere a yekɔɔ hɔ
- ○ Woman unavailable for extended period of time/Na ɔbaa no nni hɔ bere a yɛde nna kakra too nna no so
- ○ Participation was refused/ Onipa no ampene
- ○ Dwelling vacant or address is not a dwelling/ Onipa no ampene
- ○ Dwelling destroyed/Na tenabea no asɛi
- ○ Dwelling not found/Yanhu tenabea no

g) Language of interview: *Kasa a nsenmisa no kɔɔ so wɔ mu*

- ○ Ghana - English/Ghana-Brofo
- ○ Ghana - Twi/Ghana-Twi

I would like to ask you some questions about your experience during childbirth and how you and your baby have been since then. You may find some of these questions very personal. However, your answers are crucial for helping to understand the condition of women in Ghana.

Let me assure you that your answers are completely confidential and will not be told to anyone, and no one else in your household will know that you were asked these questions. If I ask you any questions you don't want to answer, just let me know and I will go on to the next question.

Mepɛ sɛ mebisa wo nsɛm bi a ɛfa nea wofaa mu ɛbere a worekɔ wo ɛne sɛnea wone wo ba no ho te seesei. Nsɛmisa no bi bɛfa wabrabɔ ho nanso wo mmuae no bɛboa mmaa a ɛwɔ Ghana nyinaa

Mepɛ sɛ me hyɛ wo bɔ sɛ yɛbɛkora mmuae a wode bɛma yɛn no yie na mpo obi a ɔte wo fie rente nsɛm a yebisaa wo no. Sɛ me bisa wo asɛm bi na wompɛ sɛ wobɛma me mmuae a, ka kyere me, na yɛntoa so.

General

2. a) How old are you? ***Wadi mfe sɛn?***
years

____

b) What is your current marital status? ***Wa ware mu nsɛm te sɛn?***

- ◯ Never married/single /Menwaree da/Meyɛ sugyani
- ◯ Currently married/Seesei maware
- ◯ Separated/Yɛn ntam atete kakra
- ◯ Divorced/Magyae aware
- ◯ Widowed/Meho kani aka baabi
- ◯ Cohabiting/Onyɛɛ me ho ade nanso mene no na ɛte
- ◯ Other/Afofro
- ◯ Unknown/Mennim

c) What is your highest level of formal education? ***Wokɔɔ sukuu duruu mpɛnpɛnso bɛn?***

- ◯ No education/Mankɔ sukuu
- ◯ Pre-primary education/mfitiase sukuu
- ◯ Incomplete primary education/Manwie mfitiase sukuu
- ◯ Complete primary education/Mewiee mfitiase sukuu
- ◯ Incomplete secondary education/Manwie ntoaso sukuu
- ◯ Complete secondary education/Mewiee ntoaso sukuu
- ◯ Incomplete post-secondary/tertiary education /Mewiee ntoaso sukuu ɛsan toa so kakra nanso manwie
- ◯ Complete post-secondary/tertiary education /Mewiee ntoaso sukuu /Suapon
- ◯ Other/Afofro
- ◯ Vocational training/Mekɔɔ sukuu kosuaa nsaano adwuma
- ◯ Unknown/Mennim

If Other, specify: ***Sɛ ɛyɛ afofro a twerɛ:***

____

d) Approximately how many minutes' drive is your house from the hospital where you gave birth? ***Se wode wani kaakae a, dɔnwhere sɛn na kaa bɛtumi de afiri wo fie akodu asopiti a wokɔ woe no?***
minutes

____

e) How many times have you been pregnant? (Note to data collector: Including times you did not give birth to the baby/babies) (gravidity) ***Mpre ahe na wafa afuro da***
99 if unknown

____

f) How many times have you given birth to a baby (that lived or died) 7 months of pregnancy? ***Mperɛ dodoɔ sɛn na wawoɔ a na nyinsen no adi abosome nson a wonyaa abofra no anaa wanya abofra no?***

99 if unknown

____

g) How many children have you given birth to who are now alive? (including most recent childbirth) ***Woba a wokuta no yi ka ho a, wo mma sɛn na ɛte ase?***

99 if unknown

____

h) For your most recent childbirth, by what method did you give birth? ***Okwan bɛn so na wofa de woo woba a okura wo yi***

- ◯ Vaginal delivery/Mekyem woe
- ◯ Caesarean section/Yɛyɛɛ me operation
- ◯ Don't know/Mennim

3. a) Are you currently breastfeeding? ***Wodaso ma wo ba no nufoɔ?***

- ◯ No/Dabi
- ◯ Yes/Aane
- ◯ Unknown/Mennim

b) If No, did you ever breastfeed your baby? ***Sɛ dabi a, wama woba no nofoɔ da?***

- ◯ No/Dabi
- ◯ Yes/Aane
- ◯ Unknown/Mennim

c) How soon after birth did you start breastfeeding? ***Sɛ aane a, dɔnhwere sɛn na ediiɛ ansana worema woba no nufoɔ?***

- ◯ Within 1 hour/Dɔnhwere baako
- ◯ Within 24 hours/Dɔnhwere aduonu nnan mu
- ◯ Within 1 week/Wo nnawɔtwe baako mu
- ◯ Longer than 1 week/Bɛyɛ nnawɔtwe baako akyi
- ◯ Unknown/Mennim

MISTREATMENT

Some women tell us when they give birth they are mistreated, or treated with disrespect while in the hospital. We would like to know how common this problem is, so we would like to ask you your own experiences with childbirth. There are no right or wrong answers to these questions. It is only important to us that we understand your experiences. Nothing you tell us will be linked to your name, your children's names, or the ability of you or your family members to access health care in the future.

Some of these questions may be upsetting or stressful. As I said before, you can skip any question you are not comfortable answering, and you can stop the interview at any point.

Mmaa bi ka se sɛ wo kɔwo wɔ asopiti a, wɔtaa yɛ wɔn ayayadeɛ, anaa yɛ bu wɔn animtia.Mepe sɛ mebisa se wo nso wofaa bi mu saa ɛbere a wo woyɛ no? Mmuae a wobɛ ma biara ye.Ehia sɛ yɛbɛhu nea wofaa mu.Yɛmfa wo din anaa wo ba bi din mmata mmuaɛbi a worema yɛn ho, anaa entumi nsi wone wabusuafoɔ ho kwan sɛ wobɛkɔ asopiti daakye.

Nsɛmisa no bi bɛyɛ wo abufuo kakra nanso wowɔ akwanya se wobɛ kwati wɔn se wompɛ sɛ wobe bua

Physical Abuse

4. At any time during your time in the hospital, did any of the following events occur? ***Bere a na wowɔ asopiti no, saa nsɛm a edidi so yi bi kɔɔ so?***

a) 1. You were pinched by a health worker or other staff? ***Adwumayefo a wɔwɔ ha no bi ati wo da?***

- No/Dabi
- Yes/Aane
- Refuse/Wampene
- Don't know/Unknown /(Mennim)/Yennim

2. How many times did this happen? ***Mpen dodoɔ sen na yei kɔɔ so?***

- Once/Preko
- Twice/Mprenu
- Three or more times /Mprensa anaa eboro saa
- Don't know/Unknown /(Mennin) Yennim

3. When did this happen? ***Bere ben na yei kɔɔ so?***

- Intrapartum (from when you arrived at the hospital until the baby came out) /Efiri bere a wobaa asopiti kosi bere a wo woyɛ
- Postpartum (from when the baby came out until you left the hospital) /Efiri bere a wo woyɛ kosii bere a wofii asopiti ha
- Both intrapartum and postpartum/Bere a ma awoɔ akawo ene awoɔ no akyi
- Don't know/Unknown /(Mennim)/Onnim

4. Who did this to you? ***Hwan na ode saa yɛɛ wo?***

- Doctor/Dokota
- Midwife or Nurse/Neese
- Trainees (Medical student, Nursing, or midwifery student)/Obi a ɔwɔ nteteɛ ase
- Non-clinical staff (cleaner, administrator, other)/Obi foforo a ɔyɛ adwuma wɔ h
- Unknown/Mennim

b) 1. You were kicked by a health worker or other staff? ***Obi de ne nan bɔɔ wo?***

- No/Dabi
- Yes/Aane
- Refuse/Wampene
- Don't know/Unknown /(Mennim)/Yennim

2. How many times did this happen? ***Mprɛ dodoɔ sɛn na yei kɔɔ so?***

- Once/Preko
- Twice/Mprenu
- Three or more times /Mprensa anaa eboro saa
- Don't know/Unknown /(Mennin) Yennim

3. When did this happen? ***Bere ben na yei kɔɔ so?***

- Intrapartum (from when you arrived at the hospital until the baby came out) /Efiri bere a wobaa asopiti kosi bere a wo woyɛ
- Postpartum (from when the baby came out until you left the hospital) /Efiri bere a wo woyɛ kosii bere a wofii asopiti ha
- Both intrapartum and postpartum/Bere a ma awoɔ akawo ene awoɔ no akyi
- Don't know/Unknown /(Mennim)/Onnim

4. Who did this to you? ***Hwan na ɔyɛɛ wo saa***

- Doctor/Dokota
- Midwife or Nurse/Neese
- Trainees (Medical student, Nursing, or midwifery student)/Obi a ɔwɔ ntetеɛ ase
- Non-clinical staff (cleaner, administrator, other)/Obi foforo a ɔyɛ adwuma wɔ h
- Unknown/Mennim

c) 1. You were slapped by a health worker or other staff? ***Obi de ne nsa yam bɔɔ wo?***

- No/Dabi
- Yes/Aane
- Refuse/Wampene
- Don't know/Unknown /(Mennim)/Yennim

2. How many times did this happen? ***Mpen dodoɔ sen na yei kɔɔ so?***

- Once/Preko
- Twice/Mprenu
- Three or more times /Mprensa anaa eboro saa
- Don't know/Unknown /(Mennin) Yennim

3. When did this happen? ***Bere ben na yei kɔɔ so?***

- Intrapartum (from when you arrived at the hospital until the baby came out) /Efiri bere a wobaa asopiti kosi bere a wo woyɛ
- Postpartum (from when the baby came out until you left the hospital) /Efiri bere a wo woyɛ kosii bere a wofii asopiti ha
- Both intrapartum and postpartum/Bere a ma awoɔ akawo ene awoɔ no akyi
- Don't know/Unknown /(Mennim)/Onnim

4. Who did this to you? *Hwan na ɔyɛɛ wo saa*

- ☐ Doctor/Dokota
- ☐ Midwife or Nurse/Neese
- ☐ Trainees (Medical student, Nursing, or midwifery student)/Obi a ɔwɔ nteteɛ ase
- ☐ Non-clinical staff (cleaner, administrator, other)/Obi foforo a ɔyɛ adwuma wɔ h
- ☐ Unknown/Mennim

d) 1. You were punched by a health worker or other staff? ***Obi de twɛdeɛ bɔɔ wo?***

- ○ No/Dabi
- ○ Yes/Aane
- ○ Refuse/Wampene
- ○ Don't know/Unknown /(Mennim)/Yennim

2. How many times did this happen? *Mpen dodoɔ sen na yei kɔɔ so?*

- ○ Once/Preko
- ○ Twice/Mprenu
- ○ Three or more times /Mprensa anaa eboro saa
- ○ Don't know/Unknown /(Mennin) Yennim

3. When did this happen? *Bere ben na yei kɔɔ so?*

- ○ Intrapartum (from when you arrived at the hospital until the baby came out) /Efiri bere a wobaa asopiti kosi bere a wo woyɛ
- ○ Postpartum (from when the baby came out until you left the hospital) /Efiri bere a wo woyɛ kosii bere a wofii asopiti ha
- ○ Both intrapartum and postpartum/Bere a ma awoɔ akawo ene awoɔ no akyi
- ○ Don't know/Unknown /(Mennim)/Onnim

4. Who did this to you? *Hwan na ɔyɛɛ wo saa*

- ☐ Doctor/Dokota
- ☐ Midwife or Nurse/Neese
- ☐ Trainees (Medical student, Nursing, or midwifery student)/Obi a ɔwɔ nteteɛ ase
- ☐ Non-clinical staff (cleaner, administrator, other)/Obi foforo a ɔyɛ adwuma wɔ h
- ☐ Unknown/Mennim

e) 1. You were hit with an instrument by a health worker or other staff? ***Obi de nneɛma a yɛde yɛ adwuma wo asopiti no bi bɔɔ wo?***

- ○ No/Dabi
- ○ Yes/Aane
- ○ Refuse/Wampene
- ○ Don't know/Unknown /(Mennim)/Yennim

2. How many times did this happen? ***Mpen dodoɔ sen na yei kɔɔ so?***

- Once/Preko
- Twice/Mprenu
- Three or more times /Mprensa anaa eboro saa
- Don't know/Unknown /(Mennin) Yennim

3. When did this happen? ***Bere ben na yei kɔɔ so?***

- Intrapartum (from when you arrived at the hospital until the baby came out) /Efiri bere a wobaa asopiti kosi bere a wo woyɛ
- Postpartum (from when the baby came out until you left the hospital) /Efiri bere a wo woyɛ kosii bere a wofii asopiti ha
- Both intrapartum and postpartum/Bere a ma awoɔ akawo ene awoɔ no akyi
- Don't know/Unknown /(Mennim)/Onnim

4. Who did this to you? ***Hwan na ɔyɛɛ wo saa***

- Doctor/Dokota
- Midwife or Nurse/Neese
- Trainees (Medical student, Nursing, or midwifery student)/Obi a ɔwɔ ntetеɛ ase
- Non-clinical staff (cleaner, administrator, other)/Obi foforo a ɔyɛ adwuma wɔ h
- Unknown/Mennim

f) 1. You were gagged (eg: something put across or in your mouth to prevent you from speaking or making noise) by a health worker or other staff? ***Obi de biribi hyɛɛ wanum sɛnea ɛbɛyɛ na wontumi nkasa anaa nteatea mu?***

- No/Dabi
- Yes/Aane
- Refuse/Wampene
- Don't know/Unknown /(Mennim)/Yennim

2. How many times did this happen? ***Mpen dodoɔ sen na yei kɔɔ so?***

- Once/Preko
- Twice/Mprenu
- Three or more times /Mprensa anaa eboro saa
- Don't know/Unknown /(Mennin) Yennim

3. When did this happen? ***Bere ben na yei kɔɔ so***

- Intrapartum (from when you arrived at the hospital until the baby came out) /Efiri bere a wobaa asopiti kosi bere a wo woyɛ
- Postpartum (from when the baby came out until you left the hospital) /Efiri bere a wo woyɛ kosii bere a wofii asopiti ha
- Both intrapartum and postpartum/Bere a ma awoɔ akawo ene awoɔ no akyi
- Don't know/Unknown /(Mennim)/Onnim

4. Who did this to you? ***Hwan na ɔyɛɛ wo saa?***

- [ ] Doctor/Dokota
- [ ] Midwife or Nurse/Neese
- [ ] Trainees (Medical student, Nursing, or midwifery student)/Obi a ɔwɔ ntetеɛ ase
- [ ] Non-clinical staff (cleaner, administrator, other)/Obi foforo a ɔyɛ adwuma wɔ h
- [ ] Unknown/Mennim

g) 1. You were physically tied to the bed by a health worker or other staff? ***Apomuden ho adwumayefo no bi kyekyeree wo bɔɔ mpa no ho?***

- ◯ No/Dabi
- ◯ Yes/Aane
- ◯ Refuse/Wampene
- ◯ Don't know/Unknown /(Mennim)/Yennim

2. How many times did this happen? ***Mpen dodoɔ sen na yei kɔɔ so?***

- ◯ Once/Preko
- ◯ Twice/Mprenu
- ◯ Three or more times /Mprensa anaa eboro saa
- ◯ Don't know/Unknown /(Mennin) Yennim

3. When did this happen? ***Bere ben na yei kɔɔ so?)***

- ◯ Intrapartum (from when you arrived at the hospital until the baby came out) /Efiri bere a wobaa asopiti kosi bere a wo woyɛ
- ◯ Postpartum (from when the baby came out until you left the hospital) /Efiri bere a wo woyɛ kosii bere a wofii asopiti ha
- ◯ Both intrapartum and postpartum/Bere a ma awoɔ akawo ene awoɔ no akyi
- ◯ Don't know/Unknown /(Mennim)/Onnim

4. Who did this to you? ***Hwan na ɔyɛɛ wo saa***

- [ ] Doctor/Dokota
- [ ] Midwife or Nurse/Neese
- [ ] Trainees (Medical student, Nursing, or midwifery student)/Obi a ɔwɔ ntetеɛ ase
- [ ] Non-clinical staff (cleaner, administrator, other)/Obi foforo a ɔyɛ adwuma wɔ h
- [ ] Unknown/Mennim

h) 1. You were held down to the bed forcefully by a health worker or other staff? ***Apomuden ho adwumayefo no bi miaa wo so wɔ mpa no so?***

- ◯ No/Dabi
- ◯ Yes/Aane
- ◯ Refuse/Wampene
- ◯ Don't know/Unknown /(Mennim)/Yennim

2. How many times did this happen? ***Mpen dodoɔ sen na yei kɔɔ so?***

- Once/Preko
- Twice/Mprenu
- Three or more times /Mprensa anaa eboro saa
- Don't know/Unknown /(Mennin) Yennim

3. When did this happen? ***Bere ben na yei kɔɔ so?***

- Intrapartum (from when you arrived at the hospital until the baby came out) /Efiri bere a wobaa asopiti kosi bere a wo woyɛ
- Postpartum (from when the baby came out until you left the hospital) /Efiri bere a wo woyɛ kosii bere a wofii asopiti ha
- Both intrapartum and postpartum/Bere a ma awoɔ akawo ene awoɔ no akyi
- Don't know/Unknown /(Mennim)/Onnim

4. Who did this to you? ***Hwan na ɔyɛɛ wo saa***

- Doctor/Dokota
- Midwife or Nurse/Neese
- Trainees (Medical student, Nursing, or midwifery student)/Obi a ɔwɔ ntetɛɛ ase
- Non-clinical staff (cleaner, administrator, other)/Obi foforo a ɔyɛ adwuma wɔ h
- Unknown/Mennim

i) 1. You had forceful downward pressure placed on your abdomen before the baby came out (fundal pressure)? ***Yemiaa wafuru so ansana abofra no reba***

- No/Dabi
- Yes/Aane
- Refuse/Wampene
- Don't know/Unknown /(Mennim)/Yennim

2. How many times did this happen? ***Mpen dodoɔ sen na yei kɔɔ so?***

- Once/Preko
- Twice/Mprenu
- Three or more times /Mprensa anaa eboro saa
- Don't know/Unknown /(Mennin) Yennim

3. When did this happen? ***Bere ben na yei kɔɔ so?***

- Intrapartum (from when you arrived at the hospital until the baby came out) /Efiri bere a wobaa asopiti kosi bere a wo woyɛ
- Postpartum (from when the baby came out until you left the hospital) /Efiri bere a wo woyɛ kosii bere a wofii asopiti ha
- Both intrapartum and postpartum/Bere a ma awoɔ akawo ene awoɔ no akyi
- Don't know/Unknown /(Mennim)/Onnim

4. Who did this to you? ***Hwan na ɔyɛɛ wo saa***

- [ ] Doctor/Dokota
- [ ] Midwife or Nurse/Neese
- [ ] Trainees (Medical student, Nursing, or midwifery student)/Obi a ɔwɔ nteteɛ ase
- [ ] Non-clinical staff (cleaner, administrator, other)/Obi foforo a ɔyɛ adwuma wɔ h
- [ ] Unknown/Mennim

j) 1. You had another form of physical force used against you (please specify)? ***Apomuden Adwumayefo no bi de ɔhyɛ too wo so ansana worewo (Deɛ ɛwo he)?***

- ◯ No/Dabi
- ◯ Yes/Aane
- ◯ Refuse/Wampene
- ◯ Don't know/Unknown /(Mennim)/Yennim

If Other, specify: ***Sɛ afoforo bi a, kyerɛ***

____

2. How many times did this happen? ***Mpen dodoɔ sen na yei kɔɔ so?***

- ◯ Once/Preko
- ◯ Twice/Mprenu
- ◯ Three or more times /Mprensa anaa eboro saa
- ◯ Don't know/Unknown /(Mennin) Yennim

3. When did this happen? ***Bere ben na yei kɔɔ so?***

- ◯ Intrapartum (from when you arrived at the hospital until the baby came out) /Efiri bere a wobaa asopiti kosi bere a wo woyɛ
- ◯ Postpartum (from when the baby came out until you left the hospital) /Efiri bere a wo woyɛ kosii bere a wofii asopiti ha
- ◯ Both intrapartum and postpartum/Bere a ma awoɔ akawo ene awoɔ no akyi
- ◯ Don't know/Unknown /(Mennim)/Onnim

4. Who did this to you? ***Hwan na ɔyɛɛ wo saa***

- [ ] Doctor/Dokota
- [ ] Midwife or Nurse/Neese
- [ ] Trainees (Medical student, Nursing, or midwifery student)/Obi a ɔwɔ nteteɛ ase
- [ ] Non-clinical staff (cleaner, administrator, other)/Obi foforo a ɔyɛ adwuma wɔ h
- [ ] Unknown/Mennim

Verbal Abuse

5. During your time in the hospital, did any of the following events occur? ***Bere a na wowɔ asopiti no, nea edidi so yi bi kɔɔ so?***

a) 1. You were shouted or screamed at by a health worker or other staff? ***Apomuden ho adwumayefo no bi teaa wo?***

- No/Dabi
- Yes/Aane
- Refuse/Wampene
- Don't know/Unknown /(Mennim)/Yennim

2. How many times did this happen? ***Mpen dodoɔ sen na yei kɔɔ so?***

- Once/Preko
- Twice/Mprenu
- Three or more times /Mprensa anaa eboro saa
- Don't know/Unknown /(Mennin) Yennim

3. When did this happen? ***Bere ben na yei kɔɔ so?***

- Intrapartum (from when you arrived at the hospital until the baby came out) /Efiri bere a wobaa asopiti kosi bere a wo woyɛ
- Postpartum (from when the baby came out until you left the hospital) /Efiri bere a wo woyɛ kosii bere a wofii asopiti ha
- Both intrapartum and postpartum/Bere a ma awoɔ akawo ene awoɔ no akyi
- Don't know/Unknown /(Mennim)/Onnim

4. Who did this to you? ***Hwan na ɔyɛɛ wo saa***

- Doctor/Dokota
- Midwife or Nurse/Neese
- Trainees (Medical student, Nursing, or midwifery student)/Obi a ɔwɔ nteteɛ ase
- Non-clinical staff (cleaner, administrator, other)/Obi foforo a ɔyɛ adwuma wɔ h
- Unknown/Mennim

b) 1. You were insulted by a health worker or other staff? ***Apɔmuden Adwumayefo bi dii wo atɛm?***

- No/Dabi
- Yes/Aane
- Refuse/Wampene
- Don't know/Unknown /(Mennim)/Yennim

2. How many times did this happen? ***Mpen dodoɔ sen na yei kɔɔ so?***

- ◯ Once/Preko
- ◯ Twice/Mprenu
- ◯ Three or more times /Mprensa anaa eboro saa
- ◯ Don't know/Unknown /(Mennin) Yennim

3. When did this happen? ***Bere ben na yei kɔɔ so?***

- ◯ Intrapartum (from when you arrived at the hospital until the baby came out) /Efiri bere a wobaa asopiti kosi bere a wo woyɛ
- ◯ Postpartum (from when the baby came out until you left the hospital) /Efiri bere a wo woyɛ kosii bere a wofii asopiti ha
- ◯ Both intrapartum and postpartum/Bere a ma awoɔ akawo ene awoɔ no akyi
- ◯ Don't know/Unknown /(Mennim)/Onnim

4. Who did this to you? ***Hwan na ɔyɛɛ wo saa?***

- ☐ Doctor/Dokota
- ☐ Midwife or Nurse/Neese
- ☐ Trainees (Medical student, Nursing, or midwifery student)/Obi a ɔwɔ nteteɛ ase
- ☐ Non-clinical staff (cleaner, administrator, other)/Obi foforo a ɔyɛ adwuma wɔ h
- ☐ Unknown/Mennim

c) 1. You were scolded by a health worker or other staff? ***Apomuden Adwumayefo bi kaa wanim?***

- ◯ No/Dabi
- ◯ Yes/Aane
- ◯ Refuse/Wampene
- ◯ Don't know/Unknown /(Mennim)/Yennim

2. How many times did this happen? ***Mpen dodoɔ sen na yei kɔɔ so?***

- ◯ Once/Preko
- ◯ Twice/Mprenu
- ◯ Three or more times /Mprensa anaa eboro saa
- ◯ Don't know/Unknown /(Mennin) Yennim

3. When did this happen? ***Bere ben na yei kɔɔ so?***

- ◯ Intrapartum (from when you arrived at the hospital until the baby came out) /Efiri bere a wobaa asopiti kosi bere a wo woyɛ
- ◯ Postpartum (from when the baby came out until you left the hospital) /Efiri bere a wo woyɛ kosii bere a wofii asopiti ha
- ◯ Both intrapartum and postpartum/Bere a ma awoɔ akawo ene awoɔ no akyi
- ◯ Don't know/Unknown /(Mennim)/Onnim

4. Who did this to you? ***Hwan na ɔyɛɛ wo saa***

- ☐ Doctor/Dokota
- ☐ Midwife or Nurse/Neese
- ☐ Trainees (Medical student, Nursing, or midwifery student)/Obi a ɔwɔ nteteɛ ase
- ☐ Non-clinical staff (cleaner, administrator, other)/Obi foforo a ɔyɛ adwuma wɔ h
- ☐ Unknown/Mennim

d) 1. You were mocked by a health worker or other staff? ***Apomuden mu Adwumayefo bi dii wo ho fɛw***

- ◯ No/Dabi
- ◯ Yes/Aane
- ◯ Refuse/Wampene
- ◯ Don't know/Unknown /(Mennim)/Yennim

2. How many times did this happen? ***Mpen dodoɔ sen na yei kɔɔ so?***

- ◯ Once/Preko
- ◯ Twice/Mprenu
- ◯ Three or more times /Mprensa anaa eboro saa
- ◯ Don't know/Unknown /(Mennin) Yennim

3. When did this happen? ***Bere ben na yei kɔɔ so?***

- ◯ Intrapartum (from when you arrived at the hospital until the baby came out) /Efiri bere a wobaa asopiti kosi bere a wo woyɛ
- ◯ Postpartum (from when the baby came out until you left the hospital) /Efiri bere a wo woyɛ kosii bere a wofii asopiti ha
- ◯ Both intrapartum and postpartum/Bere a ma awoɔ akawo ene awoɔ no akyi
- ◯ Don't know/Unknown /(Mennim)/Onnim

4. Who did this to you? ***Hwan na ɔyɛɛ wo saa***

- ☐ Doctor/Dokota
- ☐ Midwife or Nurse/Neese
- ☐ Trainees (Medical student, Nursing, or midwifery student)/Obi a ɔwɔ nteteɛ ase
- ☐ Non-clinical staff (cleaner, administrator, other)/Obi foforo a ɔyɛ adwuma wɔ h
- ☐ Unknown/Mennim

e) 1. The health worker or other staff member made negative comments about your physical appearance (such as your weight, private parts, cleanliness or other parts of your body)? ***Apomuden mu Adwumayefo bi kaa nsem a ɛnyɛ de wo senea wo kese,wanam mu anaa wahote ho?***

- ◯ No/Dabi
- ◯ Yes/Aane
- ◯ Refuse/Wampene
- ◯ Don't know/Unknown /(Mennim)/Yennim

2. How many times did this happen? ***Mpen dodoɔ sen na yei kɔɔ so?***

- ◯ Once/Preko
- ◯ Twice/Mprenu
- ◯ Three or more times /Mprensa anaa eboro saa
- ◯ Don't know/Unknown /(Mennin) Yennim

3. When did this happen? ***Bere ben na yei kɔɔ so?***

- ◯ Intrapartum (from when you arrived at the hospital until the baby came out) /Efiri bere a wobaa asopiti kosi bere a wo woyɛ
- ◯ Postpartum (from when the baby came out until you left the hospital) /Efiri bere a wo woyɛ kosii bere a wofii asopiti ha
- ◯ Both intrapartum and postpartum/Bere a ma awoɔ akawo ene awoɔ no akyi
- ◯ Don't know/Unknown /(Mennim)/Onnim

4. Who did this to you? ***Hwan na ɔyɛɛ wo saa***

- ☐ Doctor/Dokota
- ☐ Midwife or Nurse/Neese
- ☐ Trainees (Medical student, Nursing, or midwifery student)/Obi a ɔwɔ ntetɛɛ ase
- ☐ Non-clinical staff (cleaner, administrator, other)/Obi foforo a ɔyɛ adwuma wɔ h
- ☐ Unknown/Mennim

f) 1. The health worker or other staff member made negative comments about your baby's physical appearance (such as his/her appearance, sex or other aspects of the baby)? ***Apomuden mu Adwumayefo bi kaa nsɛm bi a ɛnyɛ dɛ faa sɛnea wo ba no te anaa sɛ oyɛ ɔbarima anaa obaa?***

- ◯ No/Dabi
- ◯ Yes/Aane
- ◯ Refuse/Wampene
- ◯ Don't know/Unknown /(Mennim)/Yennim

2. How many times did this happen? ***Mpen dodoɔ sen na yei kɔɔ so?***

- ◯ Once/Preko
- ◯ Twice/Mprenu
- ◯ Three or more times /Mprensa anaa eboro saa
- ◯ Don't know/Unknown /(Mennin) Yennim

3. When did this happen? ***Bere ben na yei kɔɔ so?***

- ◯ Intrapartum (from when you arrived at the hospital until the baby came out) /Efiri bere a wobaa asopiti kosi bere a wo woyɛ
- ◯ Postpartum (from when the baby came out until you left the hospital) /Efiri bere a wo woyɛ kosii bere a wofii asopiti ha
- ◯ Both intrapartum and postpartum/Bere a ma awoɔ akawo ene awoɔ no akyi
- ◯ Don't know/Unknown /(Mennim)/Onnim

4. Who did this to you? ***Hwan na ɔyɛɛ wo saa***

- [ ] Doctor/Dokota
- [ ] Midwife or Nurse/Neese
- [ ] Trainees (Medical student, Nursing, or midwifery student)/Obi a ɔwɔ nteteɛ ase
- [ ] Non-clinical staff (cleaner, administrator, other)/Obi foforo a ɔyɛ adwuma wɔ h
- [ ] Unknown/Mennim

g) 1. The health worker or other staff member made negative comments to you regarding your sexual activity? ***Apomuden mu Adwumayefo bi kaa nsɛm bi a ɛnyɛ dɛ faa sɛnea wo ne mmarima hyia/da mu?***

- ○ No/Dabi
- ○ Yes/Aane
- ○ Refuse/Wampene
- ○ Don't know/Unknown /(Mennim)/Yennim

2. How many times did this happen? ***Mpen dodoɔ sen na yei kɔɔ so?***

- ○ Once/Preko
- ○ Twice/Mprenu
- ○ Three or more times /Mprensa anaa eboro saa
- ○ Don't know/Unknown /(Mennin) Yennim

3. When did this happen? ***Bere ben na yei kɔɔ so?***

- ○ Intrapartum (from when you arrived at the hospital until the baby came out) /Efiri bere a wobaa asopiti kosi bere a wo woyɛ
- ○ Postpartum (from when the baby came out until you left the hospital) /Efiri bere a wo woyɛ kosii bere a wofii asopiti ha
- ○ Both intrapartum and postpartum/Bere a ma awoɔ akawo ene awoɔ no akyi
- ○ Don't know/Unknown /(Mennim)/Onnim

4. Who did this to you? ***Hwan na ɔyɛɛ wo saa***

- [ ] Doctor/Dokota
- [ ] Midwife or Nurse/Neese
- [ ] Trainees (Medical student, Nursing, or midwifery student)/Obi a ɔwɔ nteteɛ ase
- [ ] Non-clinical staff (cleaner, administrator, other)/Obi foforo a ɔyɛ adwuma wɔ h
- [ ] Unknown/Mennim

h) 1. The health worker or other staff threatened you with a medical procedure (such as episiotomy or caesarean section)? ***Apomuden mu Adwumayefo bi hunahuna wo sɛ yɛbɛyɛ wo biribi te sɛ operation anaa yebɛtwa wase***

- ○ No/Dabi
- ○ Yes/Aane
- ○ Refuse/Wampene
- ○ Don't know/Unknown /(Mennim)/Yennim

2. How many times did this happen? ***Mpen dodoɔ sen na yei kɔɔ so?***

- Once/Preko
- Twice/Mprenu
- Three or more times /Mprensa anaa eboro saa
- Don't know/Unknown /(Mennin) Yennim

3. When did this happen? ***Bere ben na yei kɔɔ so?***

- Intrapartum (from when you arrived at the hospital until the baby came out) /Efiri bere a wobaa asopiti kosi bere a wo woyɛ
- Postpartum (from when the baby came out until you left the hospital) /Efiri bere a wo woyɛ kosii bere a wofii asopiti ha
- Both intrapartum and postpartum/Bere a ma awoɔ akawo ene awoɔ no akyi
- Don't know/Unknown /(Mennim)/Onnim

4. Who did this to you? ***Hwan na ɔyɛɛ wo saa***

- Doctor/Dokota
- Midwife or Nurse/Neese
- Trainees (Medical student, Nursing, or midwifery student)/Obi a ɔwɔ ntetеɛ ase
- Non-clinical staff (cleaner, administrator, other)/Obi foforo a ɔyɛ adwuma wɔ h
- Unknown/Mennim

i) 1. The health worker or other staff threatened you with physical violence? ***Apomuden mu adwumayefo bi hunahuna wo sɛ yɛbɛbo wo anaa?***

- No/Dabi
- Yes/Aane
- Refuse/Wampene
- Don't know/Unknown /(Mennim)/Yennim

2. How many times did this happen? ***Mpen dodoɔ sen na yei kɔɔ so?***

- Once/Preko
- Twice/Mprenu
- Three or more times /Mprensa anaa eboro saa
- Don't know/Unknown /(Mennin) Yennim

3. When did this happen? ***Bere ben na yei kɔɔ so?***

- Intrapartum (from when you arrived at the hospital until the baby came out) /Efiri bere a wobaa asopiti kosi bere a wo woyɛ
- Postpartum (from when the baby came out until you left the hospital) /Efiri bere a wo woyɛ kosii bere a wofii asopiti ha
- Both intrapartum and postpartum/Bere a ma awoɔ akawo ene awoɔ no akyi
- Don't know/Unknown /(Mennim)/Onnim

4. Who did this to you? ***Hwan na ɔyɛɛ wo saa***

- [ ] Doctor/Dokota
- [ ] Midwife or Nurse/Neese
- [ ] Trainees (Medical student, Nursing, or midwifery student)/Obi a ɔwɔ ntetɛɛ ase
- [ ] Non-clinical staff (cleaner, administrator, other)/Obi foforo a ɔyɛ adwuma wɔ h
- [ ] Unknown/Mennim

j) 1. The health worker or other staff threatened that if you did not comply, you or your baby would have poor outcome: ***Apomuden mu adwumayefo bi hunahuna wo sɛ, sɛ woamma yanyɛ nea yɛbɛyɛ ama wo a, wankasa anaa woba no ɛnwie papa:***

- ◯ No/Dabi
- ◯ Yes/Aane
- ◯ Refuse/Wampene
- ◯ Don't know/Unknown /(Mennim)/Yennim

2. How many times did this happen? ***Mpen dodoɔ sen na yei kɔɔ so?***

- ◯ Once/Preko
- ◯ Twice/Mprenu
- ◯ Three or more times /Mprensa anaa eboro saa
- ◯ Don't know/Unknown /(Mennin) Yennim

3. When did this happen? ***Bere ben na yei kɔɔ so?***

- ◯ Intrapartum (from when you arrived at the hospital until the baby came out) /Efiri bere a wobaa asopiti kosi bere a wo woyɛ
- ◯ Postpartum (from when the baby came out until you left the hospital) /Efiri bere a wo woyɛ kosii bere a wofii asopiti ha
- ◯ Both intrapartum and postpartum/Bere a ma awoɔ akawo ene awoɔ no akyi
- ◯ Don't know/Unknown /(Mennim)/Onnim

4. Who did this to you? ***Hwan na ɔyɛɛ wo saa***

- [ ] Doctor/Dokota
- [ ] Midwife or Nurse/Neese
- [ ] Trainees (Medical student, Nursing, or midwifery student)/Obi a ɔwɔ ntetɛɛ ase
- [ ] Non-clinical staff (cleaner, administrator, other)/Obi foforo a ɔyɛ adwuma wɔ h
- [ ] Unknown/Mennim

k) 1. The health worker or other staff threatened to withhold care from you or your baby: ***Apɔmuden odwumayeni bi hunahuna wo sɛ ɔnhwɛ wo anaa wo ba no?***

- ◯ No/Dabi
- ◯ Yes/Aane
- ◯ Refuse/Wampene
- ◯ Don't know/Unknown /(Mennim)/Yennim

2. How many times did this happen? ***Mpen dodoɔ sen na yei kɔɔ so?***

- Once/Preko
- Twice/Mprenu
- Three or more times /Mprensa anaa eboro saa
- Don't know/Unknown /(Mennin) Yennim

3. When did this happen? ***Bere ben na yei kɔɔ so?***

- Intrapartum (from when you arrived at the hospital until the baby came out) /Efiri bere a wobaa asopiti kosi bere a wo woyɛ
- Postpartum (from when the baby came out until you left the hospital) /Efiri bere a wo woyɛ kosii bere a wofii asopiti ha
- Both intrapartum and postpartum/Bere a ma awoɔ akawo ene awoɔ no akyi
- Don't know/Unknown /(Mennim)/Onnim

4. Who did this to you? ***Hwan na ɔyɛɛ wo saa***

- Doctor/Dokota
- Midwife or Nurse/Neese
- Trainees (Medical student, Nursing, or midwifery student)/Obi a ɔwɔ ntetеɛ ase
- Non-clinical staff (cleaner, administrator, other)/Obi foforo a ɔyɛ adwuma wɔ h
- Unknown/Mennim

l) 1. The health worker or other staff blamed you for something that happened to you or your baby during your time in hospital: ***Apɔmuden adwumayɛni bi bɔɔ wo soboro wo bibi a esii wɔ wawoɔ mu?***

- No/Dabi
- Yes/Aane
- Refuse/Wampene
- Don't know/Unknown /(Mennim)/Yennim

2. How many times did this happen? ***Mpen dodoɔ sen na yei kɔɔ so?***

- Once/Preko
- Twice/Mprenu
- Three or more times /Mprensa anaa eboro saa
- Don't know/Unknown /(Mennin) Yennim

3. When did this happen? ***Bere ben na yei kɔɔ so?***

- Intrapartum (from when you arrived at the hospital until the baby came out) /Efiri bere a wobaa asopiti kosi bere a wo woyɛ
- Postpartum (from when the baby came out until you left the hospital) /Efiri bere a wo woyɛ kosii bere a wofii asopiti ha
- Both intrapartum and postpartum/Bere a ma awoɔ akawo ene awoɔ no akyi
- Don't know/Unknown /(Mennim)/Onnim

4. Who did this to you? ***Hwan na ɔyɛɛ wo saa***

- [ ] Doctor/Dokota
- [ ] Midwife or Nurse/Neese
- [ ] Trainees (Medical student, Nursing, or midwifery student)/Obi a ɔwɔ nteteɛ ase
- [ ] Non-clinical staff (cleaner, administrator, other)/Obi foforo a ɔyɛ adwuma wɔ h
- [ ] Unknown/Mennim

m) 1. The health worker or other staff hissed at you during your time in hospital?*Apɔmuden mu adwumayeni bi twee wo bere a na wowɔ asopiti ho?*

- ◯ No/Dabi
- ◯ Yes/Aane
- ◯ Refuse/Wampene
- ◯ Don't know/Unknown /(Mennim)/Yennim

2. How many times did this happen? ***Mpen dodoɔ sen na yei kɔɔ so?***

- ◯ Once/Preko
- ◯ Twice/Mprenu
- ◯ Three or more times /Mprensa anaa eboro saa
- ◯ Don't know/Unknown /(Mennin) Yennim

3. When did this happen? ***Bere ben na yei kɔɔ so?***

- ◯ Intrapartum (from when you arrived at the hospital until the baby came out) /Efiri bere a wobaa asopiti kosi bere a wo woyɛ
- ◯ Postpartum (from when the baby came out until you left the hospital) /Efiri bere a wo woyɛ kosii bere a wofii asopiti ha
- ◯ Both intrapartum and postpartum/Bere a ma awoɔ akawo ene awoɔ no akyi
- ◯ Don't know/Unknown /(Mennim)/Onnim

4. Who did this to you? ***Hwan na ɔyɛɛ wo saa***

- [ ] Doctor/Dokota
- [ ] Midwife or Nurse/Neese
- [ ] Trainees (Medical student, Nursing, or midwifery student)/Obi a ɔwɔ nteteɛ ase
- [ ] Non-clinical staff (cleaner, administrator, other)/Obi foforo a ɔyɛ adwuma wɔ h
- [ ] Unknown/Mennim

n) 1. Other use of verbal abuse against you (please specify): ***obi san kaa biribi tiaa wo?***

- ◯ No/Dabi
- ◯ Yes/Aane
- ◯ Refuse/Wampene
- ◯ Don't know/Unknown /(Mennim)/Yennim

If Other, specify: ***Sɛ afoforo bi a, kyerɛ***

---

2. How many times did this happen? ***Mpen dodoɔ sen na yei kɔɔ so?***

- ◯ Once/Preko
- ◯ Twice/Mprenu
- ◯ Three or more times /Mprensa anaa eboro saa
- ◯ Don't know/Unknown /(Mennin) Yennim

3. When did this happen? ***Bere ben na yei kɔɔ so?***

- ◯ Intrapartum (from when you arrived at the hospital until the baby came out) /Efiri bere a wobaa asopiti kosi bere a wo woyɛ
- ◯ Postpartum (from when the baby came out until you left the hospital) /Efiri bere a wo woyɛ kosii bere a wofii asopiti ha
- ◯ Both intrapartum and postpartum/Bere a ma awoɔ akawo ene awoɔ no akyi
- ◯ Don't know/Unknown /(Mennim)/Onnim

4. Who did this to you? ***Hwan na ɔyɛɛ wo saa***

- ☐ Doctor/Dokota
- ☐ Midwife or Nurse/Neese
- ☐ Trainees (Medical student, Nursing, or midwifery student)/Obi a ɔwɔ ntetee ase
- ☐ Non-clinical staff (cleaner, administrator, other)/Obi foforo a ɔyɛ adwuma wɔ h
- ☐ Unknown/Mennim

Stigma and Discrimination

6. During the time in hospital, did any of the following events occur? ***Mmere a wowɔ asopiti no, nea edidi so yi bi sii?***

a) 1. A health worker or staff made negative comments to you regarding your ethnicity, race, tribe or culture: ***Adwumayefo no bi kaa nsɛm a ɛfa wa busua,baabi a wofi ɛne wa mammere ho a na enfata***

- No/Dabi
- Yes/Aane
- Refuse/Wampene
- Don't know/Unknown /(Mennim)/Yennim

2. How many times did this happen? ***Mpen dodoɔ sen na yei kɔɔ so?***

- Once/Preko
- Twice/Mprenu
- Three or more times /Mprensa anaa eboro saa
- Don't know/Unknown /(Mennin) Yennim

3. When did this happen? ***Bere ben na yei kɔɔ so?***

- Intrapartum (from when you arrived at the hospital until the baby came out) /Efiri bere a wobaa asopiti kosi bere a wo woyɛ
- Postpartum (from when the baby came out until you left the hospital) /Efiri bere a wo woyɛ kosii bere a wofii asopiti ha
- Both intrapartum and postpartum/Bere a ma awoɔ akawo ene awoɔ no akyi
- Don't know/Unknown /(Mennim)/Onnim

4. Who did this to you? ***Hwan na ɔyɛɛ wo saa***

- Doctor/Dokota
- Midwife or Nurse/Neese
- Trainees (Medical student, Nursing, or midwifery student)/Obi a ɔwɔ nteteɛ ase
- Non-clinical staff (cleaner, administrator, other)/Obi foforo a ɔyɛ adwuma wɔ h
- Unknown/Mennim

b) 1. A health worker or staff made negative comments to you regarding your religion: ***Adwumayefo no bi kaa nsɛm a emfata wɔ ɔsom a wosom ho***

- No/Dabi
- Yes/Aane
- Refuse/Wampene
- Don't know/Unknown /(Mennim)/Yennim

2. How many times did this happen? ***Mpen dodoɔ sen na yei kɔɔ so?***

- ◯ Once/Preko
- ◯ Twice/Mprenu
- ◯ Three or more times /Mprensa anaa eboro saa
- ◯ Don't know/Unknown /(Mennin) Yennim

3. When did this happen? ***Bere ben na yei kɔɔ so?***

- ◯ Intrapartum (from when you arrived at the hospital until the baby came out) /Efiri bere a wobaa asopiti kosi bere a wo woyɛ
- ◯ Postpartum (from when the baby came out until you left the hospital) /Efiri bere a wo woyɛ kosii bere a wofii asopiti ha
- ◯ Both intrapartum and postpartum/Bere a ma awoɔ akawo ene awoɔ no akyi
- ◯ Don't know/Unknown /(Mennim)/Onnim

4. Who did this to you? ***Hwan na ɔyɛɛ wo saa***

- ☐ Doctor/Dokota
- ☐ Midwife or Nurse/Neese
- ☐ Trainees (Medical student, Nursing, or midwifery student)/Obi a ɔwɔ nteteɛ ase
- ☐ Non-clinical staff (cleaner, administrator, other)/Obi foforo a ɔyɛ adwuma wɔ h
- ☐ Unknown/Mennim

c) 1. A health worker or staff made negative comments to you regarding your age: ***Apɔmuden odwumayefo bi kaa nsɛm a emfata wɔ wo mfe a wadi ho?***

- ◯ No/Dabi
- ◯ Yes/Aane
- ◯ Refuse/Wampene
- ◯ Don't know/Unknown /(Mennim)/Yennim

2. How many times did this happen? ***Mpen dodoɔ sen na yei kɔɔ so?***

- ◯ Once/Preko
- ◯ Twice/Mprenu
- ◯ Three or more times /Mprensa anaa eboro saa
- ◯ Don't know/Unknown /(Mennin) Yennim

3. When did this happen? ***Bere ben na yei kɔɔ so?***

- ◯ Intrapartum (from when you arrived at the hospital until the baby came out) /Efiri bere a wobaa asopiti kosi bere a wo woyɛ
- ◯ Postpartum (from when the baby came out until you left the hospital) /Efiri bere a wo woyɛ kosii bere a wofii asopiti ha
- ◯ Both intrapartum and postpartum/Bere a ma awoɔ akawo ene awoɔ no akyi
- ◯ Don't know/Unknown /(Mennim)/Onnim

4. Who did this to you? *Hwan na ɔyɛɛ wo saa*

- [ ] Doctor/Dokota
- [ ] Midwife or Nurse/Neese
- [ ] Trainees (Medical student, Nursing, or midwifery student)/Obi a ɔwɔ ntetɛɛ ase
- [ ] Non-clinical staff (cleaner, administrator, other)/Obi foforo a ɔyɛ adwuma wɔ h
- [ ] Unknown/Mennim

d) 1. A health worker or staff made negative comments to you regarding whether you were married or not: ***Odwumayeni bi kaa nsem bi a emfata wɔ se wonwaree anaa waware ho***

- ○ No/Dabi
- ○ Yes/Aane
- ○ Refuse/Wampene
- ○ Don't know/Unknown /(Mennim)/Yennim

2. How many times did this happen? *Mpen dodoɔ sen na yei kɔɔ so?*

- ○ Once/Preko
- ○ Twice/Mprenu
- ○ Three or more times /Mprensa anaa eboro saa
- ○ Don't know/Unknown /(Mennin) Yennim

3. When did this happen? *Bere ben na yei kɔɔ so?*

- ○ Intrapartum (from when you arrived at the hospital until the baby came out) /Efiri bere a wobaa asopiti kosi bere a wo woyɛ
- ○ Postpartum (from when the baby came out until you left the hospital) /Efiri bere a wo woyɛ kosii bere a wofii asopiti ha
- ○ Both intrapartum and postpartum/Bere a ma awoɔ akawo ene awoɔ no akyi
- ○ Don't know/Unknown /(Mennim)/Onnim

4. Who did this to you? *Hwan na ɔyɛɛ wo saa*

- [ ] Doctor/Dokota
- [ ] Midwife or Nurse/Neese
- [ ] Trainees (Medical student, Nursing, or midwifery student)/Obi a ɔwɔ ntetɛɛ ase
- [ ] Non-clinical staff (cleaner, administrator, other)/Obi foforo a ɔyɛ adwuma wɔ h
- [ ] Unknown/Mennim

e) 1. A health worker or staff made negative comments to you regarding your level of education or literacy: ***Odwumayeni bi kaa nsem bi a emfata wɔ wo sukuu a wokoe ho?***

- ○ No/Dabi
- ○ Yes/Aane
- ○ Refuse/Wampene
- ○ Don't know/Unknown /(Mennim)/Yennim

2. How many times did this happen? ***Mpen dodoɔ sen na yei kɔɔ so?***

- Once/Preko
- Twice/Mprenu
- Three or more times /Mprensa anaa eboro saa
- Don't know/Unknown /(Mennin) Yennim

3. When did this happen? ***Bere ben na yei kɔɔ so?***

- Intrapartum (from when you arrived at the hospital until the baby came out) /Efiri bere a wobaa asopiti kosi bere a wo woyɛ
- Postpartum (from when the baby came out until you left the hospital) /Efiri bere a wo woyɛ kosii bere a wofii asopiti ha
- Both intrapartum and postpartum/Bere a ma awoɔ akawo ene awoɔ no akyi
- Don't know/Unknown /(Mennim)/Onnim

4. Who did this to you? ***Hwan na ɔyɛɛ wo saa***

- Doctor/Dokota
- Midwife or Nurse/Neese
- Trainees (Medical student, Nursing, or midwifery student)/Obi a ɔwɔ ntetee ase
- Non-clinical staff (cleaner, administrator, other)/Obi foforo a ɔyɛ adwuma wɔ h
- Unknown/Mennim

f) 1. A health worker or staff made negative comments to you regarding your economic circumstances: ***Odwumayeni bi kaa nsɛm a ɛmfata ɛfa wo sikasɛm ho?***

- No/Dabi
- Yes/Aane
- Refuse/Wampene
- Don't know/Unknown /(Mennim)/Yennim

2. How many times did this happen? ***Mpen dodoɔ sen na yei kɔɔ so?***

- Once/Preko
- Twice/Mprenu
- Three or more times /Mprensa anaa eboro saa
- Don't know/Unknown /(Mennin) Yennim

3. When did this happen? ***Bere ben na yei kɔɔ so?***

- Intrapartum (from when you arrived at the hospital until the baby came out) /Efiri bere a wobaa asopiti kosi bere a wo woyɛ
- Postpartum (from when the baby came out until you left the hospital) /Efiri bere a wo woyɛ kosii bere a wofii asopiti ha
- Both intrapartum and postpartum/Bere a ma awoɔ akawo ene awoɔ no akyi
- Don't know/Unknown /(Mennim)/Onnim

4. Who did this to you? ***Hwan na ɔyɛɛ wo saa***

- [ ] Doctor/Dokota
- [ ] Midwife or Nurse/Neese
- [ ] Trainees (Medical student, Nursing, or midwifery student)/Obi a ɔwɔ ntetеɛ ase
- [ ] Non-clinical staff (cleaner, administrator, other)/Obi foforo a ɔyɛ adwuma wɔ h
- [ ] Unknown/Mennim

g) 1. A health worker or staff made negative comments to you regarding your HIV status:*Odwumayeni bi kaa nsem bi a emfata a efa wo HIV ho*

- ( ) No/Dabi
- ( ) Yes/Aane
- ( ) Refuse/Wampene
- ( ) Don't know/Unknown /(Mennim)/Yennim

2. How many times did this happen? ***Mpen dodoɔ sen na yei kɔɔ so?***

- ( ) Once/Preko
- ( ) Twice/Mprenu
- ( ) Three or more times /Mprensa anaa eboro saa
- ( ) Don't know/Unknown /(Mennin) Yennim

3. When did this happen? ***Bere ben na yei kɔɔ so?***

- ( ) Intrapartum (from when you arrived at the hospital until the baby came out) /Efiri bere a wobaa asopiti kosi bere a wo woyɛ
- ( ) Postpartum (from when the baby came out until you left the hospital) /Efiri bere a wo woyɛ kosii bere a wofii asopiti ha
- ( ) Both intrapartum and postpartum/Bere a ma awoɔ akawo ene awoɔ no akyi
- ( ) Don't know/Unknown /(Mennim)/Onnim

4. Who did this to you? ***Hwan na ɔyɛɛ wo saa***

- [ ] Doctor/Dokota
- [ ] Midwife or Nurse/Neese
- [ ] Trainees (Medical student, Nursing, or midwifery student)/Obi a ɔwɔ ntetеɛ ase
- [ ] Non-clinical staff (cleaner, administrator, other)/Obi foforo a ɔyɛ adwuma wɔ h
- [ ] Unknown/Mennim

Vaginal Examinations

7. During labour and delivery, women generally receive a vaginal examination: ***Sɛ eduru bere a mmaa rekɔwo a, mpɛn pii no yɛhwɛ sɛnea wɔn ase te:***

a) Did you receive a vaginal examination at any time in hospital? ***Obi de ne nsa hyɛɛ wase bere bi wɔ asopiti hɔ ?***

- No/Dabi
- Yes/Aane
- Refuse/Wampene
- Don't know/Unknown /(Mennim)/Yennim

b) Did the health worker explain to you why a vaginal examination was needed? ***Apɔmuden mu odwumayɛni no kyerɛɛ wo nea enti a ehia sɛ yɛhwɛ wase?***

- No/Dabi
- Yes/Aane
- Refuse/Wampene
- Don't know/Unknown /(Mennim)/Yennim

c) Did the health worker ask you permission before performing vaginal examination? ***Apɔmuden mu odwumayeni no serɛɛ kwan wɔ wo hɔ ansana na ɔhwɛɛ wase?***

- No/Dabi
- Yes/Aane
- Refuse/Wampene
- Don't know/Unknown /(Mennim)/Yennim

d) Did a staff member discuss your private information about your health in a way that others could hear? ***Odwumayeni bi kaa wokoa mu nsɛm bi a efa wapɔmuden ho a obi bɛtumi ate?***

- No/Dabi
- Yes/Aane
- Refuse/Wampene
- Don't know/Unknown /(Mennim)/Yennim

e) Were vaginal examinations conducted privately (in a way that other people could not see)? ***Bere a yɛhwɛɛ wase no, yɛyɛɛ no kokoa mu sɛ nea obi ntumi nhu?***

- No/Dabi
- Yes/Aane
- Refuse/Wampene
- Don't know/Unknown /(Mennim)/Yennim

f) In general, how would you describe your experience of these vaginal examinations? ***Ne nyinna mu no, sɛn na wobɛka afa sɛnea wase hwɛ no kɔe ho?***

- ○ Comfortable/Eye
- ○ A little uncomfortable/Ɛnyɛ kakra
- ○ Quite uncomfortable/Ɛnyɛ papa
- ○ Very uncomfortable/Ɛnyɛ koraa
- ○ Unknown/Yennim

Pain Relief

8. a) During your time in the hospital, were you offered any form of pain relief? ***Bere a wowɔ asopiti ho no, so yɛbisaa wosɛ yɛnma wo aduro a ɛbɛma ɔyaw no ano abrɛ ase?***

- ○ No/Dabi
- ○ Yes/Aane
- ○ Refuse/Wampene
- ○ Don't know/Unknown /(Mennim)/Yennim

b) Did you request pain relief during your time in hospital? ***Bere a wowɔ asopiti hɔ no wo bisaa sɛ yɛmma wo aduro a ɛbɛma ɔyaw no abrɛ ase?***

- ○ No/Dabi
- ○ Yes/Aane
- ○ Refuse/Wampene
- ○ Don't know/Unknown /(Mennim)/Yennim

c) Did you receive pain relief during your time in hospital? ***Yɛmaa wo aduro a ɛbɛma wo yaw no abrɛ ase?***

- ○ No/Dabi
- ○ Yes/Aane
- ○ Refuse/Wampene
- ○ Don't know/Unknown /(Mennim)/Yennim

d) During your time in the hospital, were you denied pain relief for any reason? ***Wowɔ asopiti no wɔkyerɛɛ wo nea enti a ɛnsɛ sɛ wɔnma wo aduro a ɛbɛma wo yaw no abrɛ ase?***

- ○ No/Dabi
- ○ Yes/Aane
- ○ Refuse/Wampene
- ○ Don't know/Unknown /(Mennim)/Yennim

Mobilization, Fluids and Companion

9. During your time in hospital: ***Bere a wowɔ asopiti hɔ no***

a) Did you have easy access to water or other oral fluids? ***Wo nyaa nsuo anaa annomniɛ bi ho kwan?***

- ◯ No/Dabi
- ◯ Yes/Aane
- ◯ Refuse/Wampene
- ◯ Don't know/Unknown /(Mennim)/Yennim

b) Were you allowed to eat? ***Yɛpene ma wo didiɛ?***

- ◯ No/Dabi
- ◯ Yes/Aane
- ◯ Refuse/Wampene
- ◯ Don't know/Unknown /(Mennim)/Yennim

c) Were you told that you could walk or move around during labour? ***Yɛka kyerɛɛ wo sɛ wobɛtumi anante bere a awoɔ aka wo no?***

- ◯ No/Dabi
- ◯ Yes/Aane
- ◯ Refuse/Wampene
- ◯ Don't know/Unknown /(Mennim)/Yennim

d) Did you walk or move around during labour? ***Wo nantee anaa wotwaa wo ho kakra bere a na awoɔ aka wo?***

- ◯ No/Dabi
- ◯ Yes/Aane
- ◯ Refuse/Wampene
- ◯ Don't know/Unknown /(Mennim)/Yennim

10. a) During your time in hospital, were you allowed to have a birth companion present? For example, this includes your husband, a friend, sister, mother-in-law, etc.? ***Bere a wowɔ asopiti no yɛmaa wo kwan sɛ obi ntena wo ho?)(Sɛ ebia wase, wo kunu, anaa wadamfo?***

- ◯ No/Dabi
- ◯ Yes/Aane
- ◯ Refuse/Wampene
- ◯ Don't know/Unknown /(Mennim)/Yennim

b) 1. During your time in hospital, did you have a birth companion present at any point? ***Bere a wowɔ asopiti no yɛmaa obi kwan sɛ ɔmmra wo nkyɛn?***

- ◯ No/Dabi
- ◯ Yes/Aane
- ◯ Refuse/Wampene
- ◯ Don't know/Unknown /(Mennim)/Yennim

b) 2. If yes, who was the companion? ***Sɛ ɛyɛ aane a hwan ne nea ɔtenaa wo ho no?***

- [ ] Husband/Male partner /Kunu/Mpena
- [ ] Family member/Obusuani
- [ ] Friend/Adamfo
- [ ] Doula/Obi a ɔtena awofoɔ ho
- [ ] Traditional birth attendant/Ofie ni a ogye awoo
- [ ] Other/Afoforo

If Other, specify: ***Sɛ afoforo bi a, kyerɛ***

____

b) 3. When was the companion present? ***Bere bɛn na na onipa no wɔ wo nkyɛn?***

- ○ During labour only/Bere a awo aka me no pɛ
- ○ During childbirth only/Bere a merewo
- ○ After childbirth only/Mawo akyi nko
- ○ During labour and childbirth only/Nigba irobi ati ibimo
- ○ During labour and after childbirth only/Bere a awo aka me ne mawo akyi nko
- ○ During childbirth and after childbirth only/Bere a merewo ne mawo akyi nko
- ○ During labour, childbirth and after childbirth/Bere a awo aka me,mawo ne mawo akyi
- ○ Don't know/Unknown /Mennim/Onnim

Unreasonable Demands, Fee Structures and Neglect

11. After the birth, were you instructed to clean up your own blood, urine, faeces or amniotic fluid? ***Wawo akyi no obi hyɛɛ wo sɛ pepa wo mogya, dwonsɔ, agyanan anaa nsuo na egina woba no ho?***

- No/Dabi
- Yes/Aane
- Refuse/Wampene
- Don't know/Unknown /(Mennim)/Yennim

12. Did staff suggest or ask you (or your family or friends) for a bribe, informal payment or gift? ***Odwumayɛni bisaa wo, (wabusua, anaa wo nnanfonom) sɛ wɔmma no akyɛdeɛ bi, anaa wɔntua sika bi a na eni se otua?***

- No/Dabi
- Yes/Aane
- Refuse/Wampene
- Don't know/Unknown /(Mennim)/Yennim

13. Do you agree with the following statements? ***Wopene anaa wompene kasamu a edidi so yi?***

a) During my time in hospital for childbirth I felt ignored by the health workers or staff: ***Bere a mewɔ asopiti a merekɔwo no mefaa no sɛ adwumayefo no apo me***

- Strongly agree/Mo gba gan-an
- Agree/Mo gba
- Neutral/Mi o fi sibikan
- Disagree/Mi o gba
- Strongly disagree/Mi o gba rara
- Unknown/MI o mo

b) During my time in hospital for childbirth I felt neglected by the health workers or staff: ***Bere a mewɔ asopiti a merekɔwo no mefaa no sɛ adwumayɛfo no amma me nkyɛn***

- Strongly agree/Mo gba gan-an
- Agree/Mo gba
- Neutral/Mi o fi sibikan
- Disagree/Mi o gba
- Strongly disagree/Mi o gba rara
- Unknown/MI o mo

c) During my time in hospital for childbirth I felt that my presence was a nuisance for the health workers or staff: ***Bere a mewɔ asopiti a merekɔwo no mefaa no sɛ me ho teetee adwumayefo no***

- Strongly agree/Mo gba gan-an
- Agree/Mo gba
- Neutral/Mi o fi sibikan
- Disagree/Mi o gba
- Strongly disagree/Mi o gba rara
- Unknown/MI o mo

d) During my time in hospital for childbirth I had to wait for long periods of time before I was attended by health workers: ***Bere a mewɔ asopiti a merekɔwo no, metwɛn nnɔnhwere bebree ansana wɔrehwɛ me***

- Strongly agree/Mo gba gan-an
- Agree/Mo gba
- Neutral/Mi o fi sibikan
- Disagree/Mi o gba
- Strongly disagree/Mi o gba rara
- Unknown/MI o mo

e) During my time in hospital for childbirth I felt emotionally supported by the health workers or staff: ***Bere a mewɔ asopiti a merekɔwo no, adwumayefo no boaa me***

- Strongly agree/Mo gba gan-an
- Agree/Mo gba
- Neutral/Mi o fi sibikan
- Disagree/Mi o gba
- Strongly disagree/Mi o gba rara
- Unknown/MI o mo

f) During my time in hospital for childbirth, the health workers or staff listened to my concerns: ***Bere a mewɔ asopiti a merekowo no, adwumayefo no tiee masɛm/mehaw wɔ aso pa mu***

- Strongly agree/Mo gba gan-an
- Agree/Mo gba
- Neutral/Mi o fi sibikan
- Disagree/Mi o gba
- Strongly disagree/Mi o gba rara
- Unknown/MI o mo

g) During my time in hospital for childbirth, the health workers or staff responded to my questions or concerns: ***Bere a mewɔ asopiti a merekɔwo no, adwumayefo no yii me nsɛmmisa ne mehaw ano:***

- Strongly agree/Mo gba gan-an
- Agree/Mo gba
- Neutral/Mi o fi sibikan
- Disagree/Mi o gba
- Strongly disagree/Mi o gba rara
- Unknown/MI o mo

Childbirth

14. a) At your most recent childbirth, did you have one baby or two babies (twins)? ***Nansa yi awoɔ a wowoe no, wowoo baako anaa ntaafoɔ?***

- One baby (single birth)/ɔba baako (awoɔ baako)
- Two babies (twins, multiple birth)/ Ntafoɔ/Ntahenasa anaa nea ɛboro saa

Baby

*** 14. b) What is the sex of your baby?** ***Wowoo dɛn?***

- Male/Ɔbarima
- Female/Ɔbaa
- Unknown/Yennim

Press + if entering more than one baby (twins), or press next if entering one baby (single birth). *Mia [+]se woretwerɛ ama ntaafoɔ,anaa mia nea edi so no sɛ woretwerɛ ama abofra baako*

Childbirth1

15. a) Did you have a preferred position to give birth in? (for example, squatting on the floor, lying on your back, kneeling, on all floors, lying on your side) ***Na wowɔ ɔkwan pɔtee bi a na wopɛ sɛ anka wofa so wo? (Se ebia wobɛkoto, bɛda wakyi, wobebu nkotodwe anaa wobɛda wo nkyɛn mu)***

- No/Dabi
- Yes/Aane
- Refuse/Wampene
- Don't know/Unknown /(Mennim)/Yennim

b) Were you permitted to deliver in this position? ***Yɛpene maa wowoo wɔ ɔkwan yei so?***

- No/Dabi
- Yes/Aane
- Refuse/Wampene
- Don't know/Unknown /(Mennim)/Yennim

c) What position did you give birth in? ***Okwan bɛn so na wofa woe?***

- Dorsal/supine /Se woda wakyi na wani hwe wim
- Lithotomy/Sɛ wobɛda wakyi na wode wo nan asi mpa no so
- On all fours/Sɛ wode wo nan ne wo nsa sisi hɔ
- Squatting /Sɛ wakoto hɔ
- Sitting/Sɛ wote hɔ
- Lying on her side/Sɛ woda wo nkyɛn mu
- Other/Afofro
- Unknown/Yennim

If Other, specify: ***Sɛ afoforo bi a, kyerɛ***

____

16. a) Which health worker looked after you most of the time during your childbirth in hospital? ***Apɔmuden mu odwumayɛni bɛn na ɔhwɛɛ wo so pii bere a worekɔwo wo asopiti no?***

- No attendant/Na obiara nni hɔ
- Obstetrician/Obenfo wɔ awo mu
- Resident Doctor/Dokota
- Midwife/Awogyefoɔ no
- Nurse/Nɛɛse
- Medical Student/Osuani wo Dɔkota mu
- Midwifery Student/Osuani wo Dɔkota mu
- Other/Afoforo
- Unknown/Not Sure/Mennim/Mentumi nhu

If Other, specify: ***Sɛ afoforo bi a, kyerɛ***

---

b) During your admission for childbirth, how often was any health worker present? ***Bere a wowɔ asopiti a worekɔwo no mperɛ sɛn na na apɔmuden adwumayefo wɔ hɔ?***

- ◯ Always/Bere biara
- ◯ Most of the time/Mpɛn pii
- ◯ Some of the time/Mpɛn kakra
- ◯ Never/Koraa
- ◯ Unknown/Yennim

c) Was any health worker present when the baby came out?***Bere a na abofra no reba no na odwumayɛni bi wɔ hɔ?***

- ◯ No/Dabi
- ◯ Yes/Aane
- ◯ Refuse/Wampene
- ◯ Don't know/Unknown /(Mennim)/Yennim

17. a) During your time in hospital, did you need a language interpreter? ***Bere a wowɔ asopiti no na wohia obi sɛ ɔbɛkyerɛ wo kasa no ase?***

- ◯ No/Dabi
- ◯ Yes/Aane
- ◯ Refuse/Wampene
- ◯ Don't know/Unknown /(Mennim)/Yennim

b) Was an interpreter available? ***Na nea ɔbɛkyerɛ wo kasa no ase wɔ hɔ?***

- ◯ No/Dabi
- ◯ Yes/Aane
- ◯ Refuse/Wampene
- ◯ Don't know/Unknown /(Mennim)/Yennim

c) Who provided language interpretation for you? ***Hwan na ɔkyerɛɛ kasa no ase?***

- ◯ Professional interpreter/Obi a yatete no wɔ kasa ase kyerɛ mu
- ◯ Staff member/Apomuden dwumayeni
- ◯ Family member/Obusuani
- ◯ Another patient/Opemfo fofro
- ◯ Someone else/Obi fofro)

If Someone else, specify: ***Se obi foforo bi a, kyerɛ:***

Childbirth2

18. Were curtains, partitions, or other measures used to provide privacy for you from other patients, patients' family members, or health workers/staff not involved in providing you care? ***Yɛde ntoma twaa ɛdan no mu sɛnea ebeyɛ na obi a ɔnka wahwefoɔ no ho nhunu wo ho?***

- No/Dabi
- Yes/Aane
- Refuse/Wampene
- Don't know/Unknown /(Mennim)/Yennim

19. a) Did you have a bed to yourself during labour? ***Na w'ankasa wowɔ mpa?***

- No/Dabi
- Yes/Aane
- Refuse/Wampene
- Don't know/Unknown /(Mennim)/Yennim

b) Did you have a bed to yourself during childbirth? ***Worekɔwo na w'ankasa na wowɔ mpa?***

- No/Dabi
- Yes/Aane
- Refuse/Wampene
- Don't know/Unknown /(Mennim)/Yennim

c) Did you have a bed to yourself after you delivered but before you left the hospital (postpartum period)? ***Bere a wowoe akyi kosii se yɛ yii wo kɔɔ fie no na wowɔ mpa?***

- No/Dabi
- Yes/Aane
- Refuse/Wampene
- Don't know/Unknown /(Mennim)/Yennim

d) At any time, did you have to share a bed with another woman or women? ***Bere a na wowɔ asopiti no eduru bere bi a na wo ne obaa foforo na ɛda mpa baako so?***

- No/Dabi
- Yes/Aane
- Refuse/Wampene
- Don't know/Unknown /(Mennim)/Yennim

20. Were you or your baby detained in the hospital due to inability to pay hospital bills? ***Yɛgyee wo ne wo ba no kaa asopiti efirisɛ w'antumi antua asopiti ka no?***

- No/Dabi
- Yes/Aane
- Refuse/Wampene
- Don't know/Unknown /(Mennim)/Yennim

Maternal Interventions

21. Did you receive any of the following procedures? ***Yɛyɛɛ wo nneɛma a edidi so yi bi?***

a) 1. Was Caesarean section received? ***Nje won se ise abe fun yin lati bi omo?***

- No/Dabi
- Yes/Aane
- Refuse/Wampene
- Don't know/Unknown /(Mennim)/Yennim

2. If yes, was the procedure explained? ***Sɛ ɛyɛ aane a yɛkyerɛɛ wo ase?***

- No/Dabi
- Yes/Aane
- Refuse/Wampene
- Don't know/Unknown /(Mennim)/Yennim

3. If yes, did you agree to the procedure? ***Sɛ ɛyɛ aane a wopenee nea yɛyɛɛ wo no so***

- No/Dabi
- Yes/Aane
- Refuse/Wampene
- Don't know/Unknown /(Mennim)/Yennim

b) 1. Was Episiotomy received? ***Nje won ge ojuara yin bi?***

- No/Dabi
- Yes/Aane
- Refuse/Wampene
- Don't know/Unknown /(Mennim)/Yennim

2. If yes, was the procedure explained? ***Sɛ ɛyɛ aane a yɛkyerɛɛ wo ase?***

- No/Dabi
- Yes/Aane
- Refuse/Wampene
- Don't know/Unknown /(Mennim)/Yennim

3. If yes, did you agree to the procedure? ***Sɛ ɛyɛ aane a wopenee nea yɛyɛɛ wo no so***

- No/Dabi
- Yes/Aane
- Refuse/Wampene
- Don't know/Unknown /(Mennim)/Yennim

c) 1. Was hysterectomy received? ***Nje won gbe ile omo yin jade bi??***

- No/Dabi
- Yes/Aane
- Refuse/Wampene
- Don't know/Unknown /(Mennim)/Yennim

2. If yes, was the procedure explained? ***Sɛ ɛyɛ aane a yɛkyerɛɛ wo ase?***

- No/Dabi
- Yes/Aane
- Refuse/Wampene
- Don't know/Unknown /(Mennim)/Yennim

3. If yes, did you agree to the procedure? ***Sɛ ɛyɛ aane a wopenee nea yɛyɛɛ wo no so***

- No/Dabi
- Yes/Aane
- Refuse/Wampene
- Don't know/Unknown /(Mennim)/Yennim

d) 1. Was tubal ligation / sterilization received? ***Nje won so ile omo yin pa bi?***

- No/Dabi
- Yes/Aane
- Refuse/Wampene
- Don't know/Unknown /(Mennim)/Yennim

2. If yes, was the procedure explained? ***Sɛ ɛyɛ aane a yɛkyerɛɛ wo ase?***

- No/Dabi
- Yes/Aane
- Refuse/Wampene
- Don't know/Unknown /(Mennim)/Yennim

3. If yes, did you agree to the procedure? ***Sɛ ɛyɛ aane a wopenee nea yɛyɛɛ wo no so***

- No/Dabi
- Yes/Aane
- Refuse/Wampene
- Don't know/Unknown /(Mennim)/Yennim

e) 1. Was manual removal of placenta received? ***Nje won fi owo yo ekeji/olubi omo yin bi?***

- No/Dabi
- Yes/Aane
- Refuse/Wampene
- Don't know/Unknown /(Mennim)/Yennim

2. If yes, was the procedure explained? ***Sɛ ɛyɛ aane a yɛkyerɛɛ wo ase?***

- No/Dabi
- Yes/Aane
- Refuse/Wampene
- Don't know/Unknown /(Mennim)/Yennim

3. If yes, did you agree to the procedure? ***Sɛ ɛyɛ aane a wopenee nea yɛyɛɛ wo no so***

- No/Dabi
- Yes/Aane
- Refuse/Wampene
- Don't know/Unknown /(Mennim)/Yennim

f) 1. Was assisted vaginal delivery (e.g: forceps or vacuum extraction) received? ***Nje won lo irinse lati fa omo jade lati oju ara yin bi?***

- No/Dabi
- Yes/Aane
- Refuse/Wampene
- Don't know/Unknown /(Mennim)/Yennim

2. If yes, was the procedure explained? ***Sɛ ɛyɛ aane a yɛkyerɛɛ wo ase?***

- No/Dabi
- Yes/Aane
- Refuse/Wampene
- Don't know/Unknown /(Mennim)/Yennim

3. If yes, did you agree to the procedure? ***Sɛ ɛyɛ aane a wopenee nea yɛyɛɛ wo no so***

- No/Dabi
- Yes/Aane
- Refuse/Wampene
- Don't know/Unknown /(Mennim)/Yennim

g) 1. Was postpartum IUD? insertion received? ***Nje afi oludena oyun/coil onifihe si inu ile omo ni kete ti e bi omo tau bi?***

- No/Dabi
- Yes/Aane
- Refuse/Wampene
- Don't know/Unknown /(Mennim)/Yennim

2. If yes, was the procedure explained? ***Sɛ ɛyɛ aane a yɛkyerɛɛ wo ase?***

- No/Dabi
- Yes/Aane
- Refuse/Wampene
- Don't know/Unknown /(Mennim)/Yennim

3. If yes, did you agree to the procedure? ***Sɛ ɛyɛ aane a wopenee nea yɛyɛɛ wo no so***

- No/Dabi
- Yes/Aane
- Refuse/Wampene
- Don't know/Unknown /(Mennim)/Yennim

h) 1. Was induction of labor received? ***Nje won lo oogun tabi gbe omisoke ki irobi le bere?***

- No/Dabi
- Yes/Aane
- Refuse/Wampene
- Don't know/Unknown /(Mennim)/Yennim

2. If yes, was the procedure explained? ***Sɛ ɛyɛ aane a yɛkyerɛɛ wo ase?***

- No/Dabi
- Yes/Aane
- Refuse/Wampene
- Don't know/Unknown /(Mennim)/Yennim

3. If yes, did you agree to the procedure? ***Sɛ ɛyɛ aane a wopenee nea yɛyɛɛ wo no so***

- No/Dabi
- Yes/Aane
- Refuse/Wampene
- Don't know/Unknown /(Mennim)/Yennim

i) 1. Was augmentation of labor received? ***Nje won gbe omisoke lati se iranwo ki e le bi omo kiakia?***

- No/Dabi
- Yes/Aane
- Refuse/Wampene
- Don't know/Unknown /(Mennim)/Yennim

2. If yes, was the procedure explained? ***Sɛ ɛyɛ aane a yɛkyerɛɛ wo ase?***

- No/Dabi
- Yes/Aane
- Refuse/Wampene
- Don't know/Unknown /(Mennim)/Yennim

3. If yes, did you agree to the procedure? ***Sɛ ɛyɛ aane a wopenee nea yɛyɛɛ wo no so***

- No/Dabi
- Yes/Aane
- Refuse/Wampene
- Don't know/Unknown /(Mennim)/Yennim

j) 1. Was shaving of your pubic hair received? ***Nje won fa irun abe fun yin bi?***

- No/Dabi
- Yes/Aane
- Refuse/Wampene
- Don't know/Unknown /(Mennim)/Yennim

2. If yes, was the procedure explained? ***Sɛ ɛyɛ aane a yɛkyerɛɛ wo ase?***

- No/Dabi
- Yes/Aane
- Refuse/Wampene
- Don't know/Unknown /(Mennim)/Yennim

3. If yes, did you agree to the procedure? ***Sɛ ɛyɛ aane a wopenee nea yɛyɛɛ wo no so***

- No/Dabi
- Yes/Aane
- Refuse/Wampene
- Don't know/Unknown /(Mennim)/Yennim

k) 1. Was Enema received? ***Nje won ro omi ose to loworo si iho iyagbe yin bi?***

- No/Dabi
- Yes/Aane
- Refuse/Wampene
- Don't know/Unknown /(Mennim)/Yennim

2. If yes, was the procedure explained? ***Sɛ ɛyɛ aane a yɛkyerɛɛ wo ase?***

- No/Dabi
- Yes/Aane
- Refuse/Wampene
- Don't know/Unknown /(Mennim)/Yennim

3. If yes, did you agree to the procedure? ***Sɛ ɛyɛ aane a wopenee nea yɛyɛɛ wo no so***

- No/Dabi
- Yes/Aane
- Refuse/Wampene
- Don't know/Unknown /(Mennim)/Yennim

l) 1. Was restitching of FGM scar received? ***Nje won tun oju idabe yin ran?***

- No/Dabi
- Yes/Aane
- Refuse/Wampene
- Don't know/Unknown /(Mennim)/Yennim

2. If yes, was the procedure explained? ***Sɛ ɛyɛ aane a yɛkyerɛɛ wo ase?***

- No/Dabi
- Yes/Aane
- Refuse/Wampene
- Don't know/Unknown /(Mennim)/Yennim

3. If yes, did you agree to the procedure? ***Sɛ ɛyɛ aane a wopenee nea yɛyɛɛ wo no so***

- No/Dabi
- Yes/Aane
- Refuse/Wampene
- Don't know/Unknown /(Mennim)/Yennim

*** 22. a) Is your baby alive today? *Woba no te nkwa mu ɛnnɛ?**

- No/Dabi
- Yes/Aane
- Refuse/Wampene
- Don't know/Unknown /(Mennim)/Yennim

Press + if entering more than one baby (twins), or press next if entering one baby (single birth).*Mia [+] se woretwerɛ ama ntaafoɔ,anaa mia nea edi so no sɛ woretwerɛ ama abofra baako*

*** b) If No, was your baby alive when he/she was born?** ***Sɛ ɛyɛ dabi a, ɛbere a wo woo no no na ɔte ase?***

- No/Dabi
- Yes/Aane
- Refuse/Wampene
- Don't know/Unknown /(Mennim)/Yennim

Press + if entering more than one baby (twins), or press next if entering one baby (single birth).*Mia [+] se woretwerɛ ama ntaafoɔ,anaa mia nea edi so no sɛ woretwerɛ ama abofra baako*

*** c) If Yes, was your baby alive when you left hospital?** ***Sɛ ɛyɛ aane a, na wo ba no te ase bere a na worefiri asopiti ho?***

- No/Dabi
- Yes/Aane
- Refuse/Wampene
- Don't know/Unknown /(Mennim)/Yennim

Press + if entering more than one baby (twins), or press next if entering one baby (single birth). *Mia [+] se woretwerɛ ama ntaafoɔ,anaa mia nea edi so no sɛ woretwerɛ ama abofra baako*

Postpartum Depression

As you have recently had a baby, we would like to know how you are feeling. Over the past two weeks, how often have you been bothered by any of the following problems? ***(Wo woe nkyeɛ, yɛpɛ sɛ yɛhu senea wo ho te?) Nnawɔtwe mmienu a atwamu akɔ yi, sɛn na nneɛma a edidi so yi bi aha wo?***

23. a) Little interest or pleasure doing things: ***Wani ɛngye wɔ wadeyɛ mu:***

- ◯ Not at all/ Biribiara nhaa me
- ◯ Several days/Enna bebree
- ◯ More than half the days/Eboro nna no mu fa
- ◯ Nearly every day/ɛye aye se eda biara

b) Feeling down, depressed or hopeless: ***Waboto, Wo werɛ aho, anaa sɛ ɛyɛ wosɛ bibiara nyɛ yie:***

- ◯ Not at all/ Biribiara nhaa me
- ◯ Several days/Enna bebree
- ◯ More than half the days/Eboro nna no mu fa
- ◯ Nearly every day/ɛye aye se eda biara

c) Trouble falling asleep, staying asleep, or sleeping too much: ***Sɛ wontumi nna anaa woda dodo:***

- ◯ Not at all/ Biribiara nhaa me
- ◯ Several days/Enna bebree
- ◯ More than half the days/Eboro nna no mu fa
- ◯ Nearly every day/ɛye aye se eda biara

d) Feeling tired or having little energy: ***Se wabere anaa wonni ahoɔden:***

- ◯ Not at all/ Biribiara nhaa me
- ◯ Several days/Enna bebree
- ◯ More than half the days/Eboro nna no mu fa
- ◯ Nearly every day/ɛye aye se eda biara

e) Poor appetite or overeating: ***Se aduane nno wo kɔn anaa wodidi dodoɔ:***

- ◯ Not at all/ Biribiara nhaa me
- ◯ Several days/Enna bebree
- ◯ More than half the days/Eboro nna no mu fa
- ◯ Nearly every day/ɛye aye se eda biara

f) Feeling bad about yourself - or that you're a failure or have let yourself or your family down: ***Se wani nnye wankasa wo ho, na aye se wadi wone wabusua fo hwanmo:***

- ◯ Not at all/ Biribiara nhaa me
- ◯ Several days/Enna bebree
- ◯ More than half the days/Eboro nna no mu fa
- ◯ Nearly every day/ɛye aye se eda biara

g) Trouble concentrating on things, such as reading the newspaper or watching television: ***Sɛ wadwen ntumi nsisi nneɛma so yiye sɛ ebia worehwɛ TV anaa worekan dawubɔ krataa:***

- ◯ Not at all/ Biribiara nhaa me
- ◯ Several days/Enna bebree
- ◯ More than half the days/Eboro nna no mu fa
- ◯ Nearly every day/ɛye aye se eda biara

h) Moving or speaking so slowly that other people could have noticed. Or, the opposite - being so fidgety or restless that you have been moving around a lot more than usual: ***Wonante anaa wokasa brɛɛ a amanfo ahyɛ no nso---anaa wayɛ basaa na wo nante kyɛn sɛnea na woyɛ daa:***

- ◯ Not at all/ Biribiara nhaa me
- ◯ Several days/Enna bebree
- ◯ More than half the days/Eboro nna no mu fa
- ◯ Nearly every day/ɛye aye se eda biara

i) Thoughts that you would be better off dead or of hurting yourself in some way: ***Ɛyɛ wo sɛ wowui a anka ɛyɛ anaa sɛ wobɛ pira wo ho:***

- ◯ Not at all/ Biribiara nhaa me
- ◯ Several days/Enna bebree
- ◯ More than half the days/Eboro nna no mu fa
- ◯ Nearly every day/ɛye aye se eda biara

Future Childbearing Intentions

24. In the future, I would like to have another child: ***Mɛpɛ sɛ mɛwo bio daakye:***

- ◯ No/Dabi
- ◯ Yes/Aane
- ◯ Refuse/Wampene
- ◯ Don't know/Unknown /(Mennim)/Yennim

25. a) Based on your most recent childbirth experience in this hospital, where would you choose for your next childbirth? ***Sɛnaa yɛfaa so hwɛɛ wo wɔ asopiti ha no ɛhe na wobɛ pɛ sɛ wobɛkɔ akɔwo bio?***

- ◯ Same hospital/Asopiti ha bio
- ◯ Different hospital/Asopiti foforo
- ◯ At my home/Ɛwɔ me fie
- ◯ At somebody else's home/Ɛwɔ obi foforo fie
- ◯ Other/Afoforo
- ◯ Refuse/Wampene
- ◯ Don't know/Unknown/Mennim/Onnim

If Other, specify: ***Sɛ afoforo bi a, kyerɛ:***

____

b) Do you agree or disagree with this statement: "I would recommend this hospital to a sister or friend" ***Wogye ɔkasamu yi to mu: sɛ mɛbɔ asopiti yi ho dawuro papa akyerɛ menua anaa madamfo ama no akɔ hɔ***

- ◯ Strongly agree/Mepene so paa
- ◯ Agree/Mepene
- ◯ Neutral/Megyina mfinfini
- ◯ Disagree/Mempene so
- ◯ Strongly disagree/Mempene so koraa

Satisfaction

Do you agree or disagree with the following statement: ***Wogye saa akasamu yi to mu anaa wongye nto mu?***

26. a) "During my stay at the hospital for childbirth, I feel that I had the opportunity to discuss any concerns I had with a health worker"? ***"Megyi di sɛ bere a me kɔ woo wɔ asopiti hɔ no, menyaa akwanya ne apɔmuden mu adwumayefo kaa nneɛma biara a na ehia/eha me ho asɛm"?***

- Strongly agree/Mepene so paa
- Agree/Mepene so
- Neutral/Megyina mfinfini
- Disagree/Mempene so
- Strongly disagree/Mempene so koraa
- Refuse/Wampene
- Unknown/Don't know/Mennim/Onnim

b) "During my stay at the hospital for childbirth, I feel that I had the opportunity to discuss any preferences or requests I had with a health worker"? ***"Megyi di se bere a me kɔ woo wɔ asopiti hɔ no, menyaa akwanya ne apɔmuden mu adwumayɛfo no kaa nea mepɛ ne mabisa deɛ ho asɛm"?***

- Strongly agree/Mepene so paa
- Agree/Mepene so
- Neutral/Megyina mfinfini
- Disagree/Mempene so
- Strongly disagree/Mempene so koraa
- Refuse/Wampene
- Unknown/Don't know/Mennim/Onnim

c) "During my stay at the hospital for childbirth, I felt that the health education and information met my needs"? ***"Megyi di se berɛ a me kɔ woo wɔ asopiti hɔ no,nkyerɛkyerɛ a menyae no wiee me pɛyɛ"?***

- Strongly agree/Mepene so paa
- Agree/Mepene so
- Neutral/Megyina mfinfini
- Disagree/Mempene so
- Strongly disagree/Mempene so koraa
- Refuse/Wampene
- Unknown/Don't know/Mennim/Onnim

d) "I feel that my privacy was respected during examinations and treatments"? ***"Megye di sɛ nhwehwɛmu ne ma yaresa kɔɔ so wɔkokoa mu a na mepɛ"?***

- Strongly agree/Mepene so paa
- Agree/Mepene so
- Neutral/Megyina mfinfini
- Disagree/Mempene so
- Strongly disagree/Mempene so koraa
- Refuse/Wampene
- Unknown/Don't know/Mennim/Onnim

e) "During my stay at the hospital for childbirth, I feel that my cultural and religious needs were respected"? ***"Me kɔwoo wo asopiti no yɛde obuo ne nidie maa me som ne ma mamrɛ "?***

- Strongly agree/Mepene so paa
- Agree/Mepene so
- Neutral/Megyina mfinfini
- Disagree/Mempene so
- Strongly disagree/Mempene so koraa
- Refuse/Wampene
- Unknown/Don't know/Mennim/Onnim

f) "During my stay at the hospital for childbirth, I feel that the health workers treated me with respect"? ***"Me ko woo wo asopiti ho no,megye di se apomuden adwumayefo de obuo maa me"?***

- Strongly agree/Mepene so paa
- Agree/Mepene so
- Neutral/Megyina mfinfini
- Disagree/Mempene so
- Strongly disagree/Mempene so koraa
- Refuse/Wampene
- Unknown/Don't know/Mennim/Onnim

g) "During my stay at the hospital for childbirth, health workers informed me about decisions that they took regarding my care"? ***"Me kɔ woo wo asopiti hɔ no,apɔmuden mu adwumayefo no bɔɔ me nsusuiɛ biara a wo faayɛ a na efa mehwɛ no ho"?***

- Strongly agree/Mepene so paa
- Agree/Mepene so
- Neutral/Megyina mfinfini
- Disagree/Mempene so
- Strongly disagree/Mempene so koraa
- Refuse/Wampene
- Unknown/Don't know/Mennim/Onnim

h) “During my labour, birth and postnatal care, I feel that I made shared decisions about my care with my health worker”? ***“Awo kaa me ne mawoɔ ne mawoɔ akyi nyinaa no ɛyɛ me sɛ mekyerɛɛ madwen fa ɔkwan a wɔfa so hwɛ me no ho”?***

- Strongly agree/Mepene so paa
- Agree/Mepene so
- Neutral/Megyina mfinfini
- Disagree/Mempene so
- Strongly disagree/Mempene so koraa
- Refuse/Wampene
- Unknown/Don't know/Mennim/Onnim

i) “During my stay at the hospital for childbirth, I feel that the amount of time I had to wait before seeing a health worker was not too long”? ***“Me kɔ woo wɔ asopiti hɔ no,megye di sɛ bere a medii ansa na apɔmuden mu adwumayɛni hwɛɛ me no ankyɛ pii”***

- Strongly agree/Mepene so paa
- Agree/Mepene so
- Neutral/Megyina mfinfini
- Disagree/Mempene so
- Strongly disagree/Mempene so koraa
- Refuse/Wampene
- Unknown/Don't know/Mennim/Onnim

j) “The facility where I gave birth had adequate water”? ***“Baabi a mekɔ woe no na nsuo,anyinam ahoɔden ne ahoteɛ wɔ hɔ”?***

- Strongly agree/Mepene so paa
- Agree/Mepene so
- Neutral/Megyina mfinfini
- Disagree/Mempene so
- Strongly disagree/Mempene so koraa
- Refuse/Wampene
- Unknown/Don't know/Mennim/Onnim

k) “The facility where I gave birth had adequate cleanliness”? ***“Ne nyinaa mu no ,mani gyee nea wɔyɛ maa me no nyinaa ho wo mawoɔ mu”?***

- Strongly agree/Mepene so paa
- Agree/Mepene so
- Neutral/Megyina mfinfini
- Disagree/Mempene so
- Strongly disagree/Mempene so koraa
- Refuse/Wampene
- Unknown/Don't know/Mennim/Onnim

l) "The facility where I gave birth had adequate electricity"? ***"Beae a mewoe no na anyinam nkanea wo hɔ abere biara"?***

- Strongly agree/Mepene so paa
- Agree/Mepene so
- Neutral/Megyina mfinfini
- Disagree/Mempene so
- Strongly disagree/Mempene so koraa
- Refuse/Wampene
- Unknown/Don't know/Mennim/Onnim

m) "Overall, I am satisfied with the services I received during my stay at the hospital for childbirth"? ***"Ne nyinaa mu no ,mani gyee nea wɔyɛ maa me no nyinaa ho wo mawoɔ mu"?***

- Strongly agree/Mepene so paa
- Agree/Mepene so
- Neutral/Megyina mfinfini
- Disagree/Mempene so
- Strongly disagree/Mempene so koraa
- Refuse/Wampene
- Unknown/Don't know/Mennim/Onnim

Comment:

____

Interviewer Code: ***Nsemisa fo no ahyɛnsode***

____

Date Community Survey Tool completed: ***Da a mpɔtamu nsemisa/nhwehwemu no baa awieɛ***

yyyy-mm-dd

Time Community Survey Tool completed: ***Bere a mpɔtamu nsemisa/nhwehwɛmu no baa awieɛ***
(00:00-23:59)

____
