# Supplementary figures and images for: Methodological development of tools to measure how women are treated during facility-based childbirth in four countries: labor observation and community survey
Source: BMC Med Res Methodol. 2018 Nov 15;18:132. doi: 10.1186/s12874-018-0603-x (PMC6238369; doi:10.1186/s12874-018-0603-x)

**Appendix 3. Screening log for the labor observation tool**


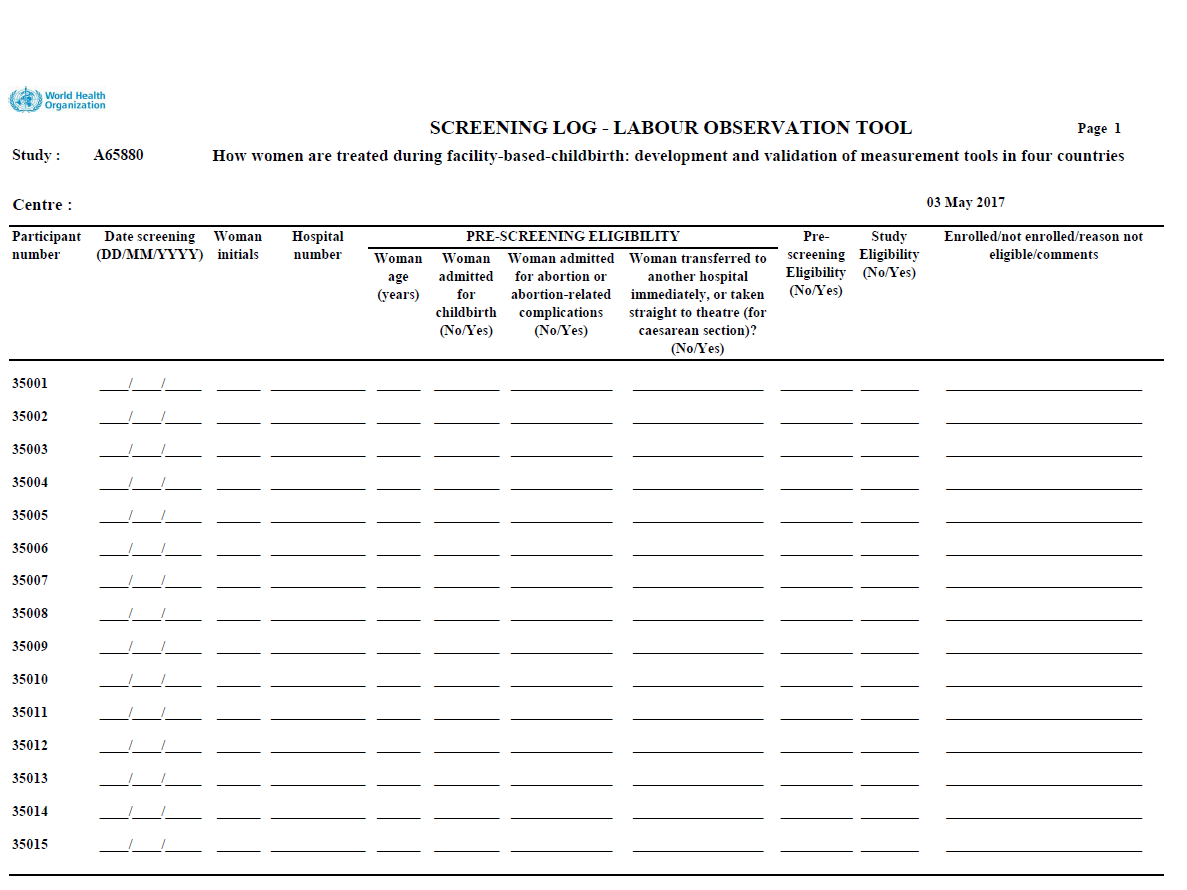

Supplement: Supplementary file 3 — Screening log for the labor observations. This is an example of the participant screening log used for the labor observation. (DOCX 96 kb) [file 12874_2018_603_MOESM3_ESM.docx]

**Appendix 4. Screening log for the community survey tool**


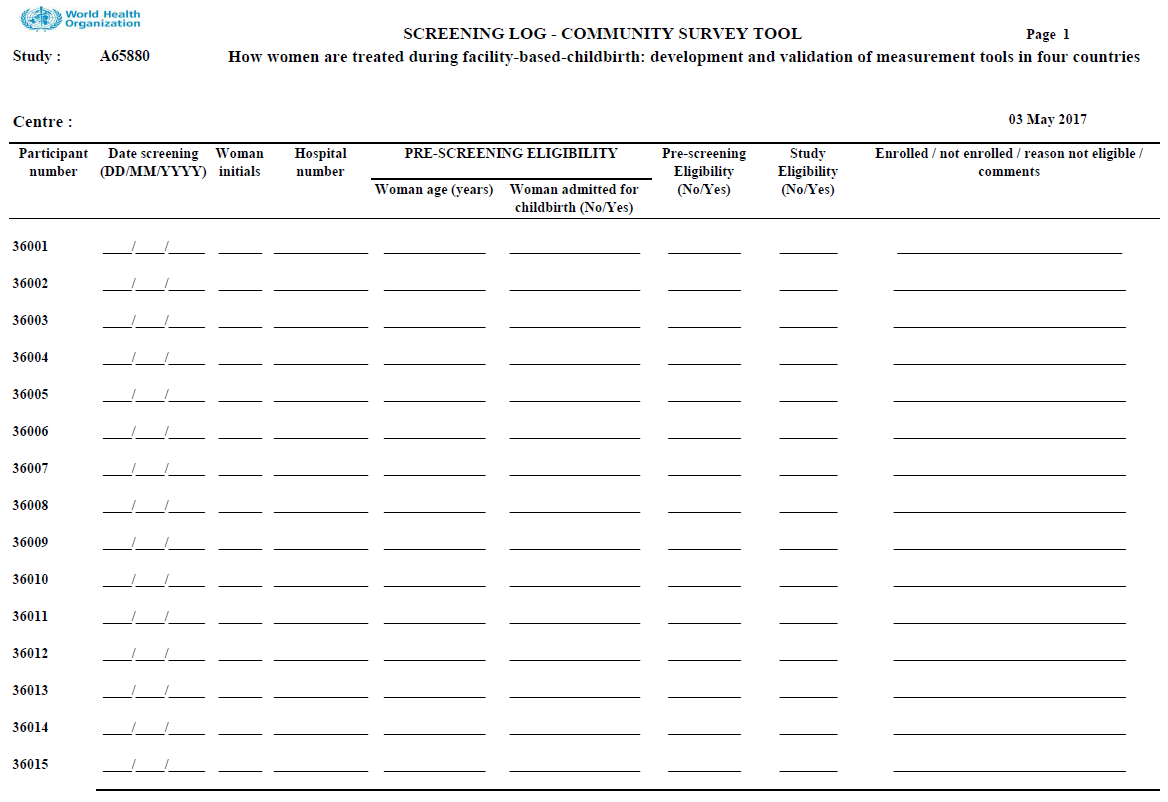

Supplement: Supplementary file 4 — Screening log for the community survey. This is an example of the participant screening log used for the community survey. (DOCX 83 kb) [file 12874_2018_603_MOESM4_ESM.docx]

**Appendix 5. Data submission log for the labor observation**


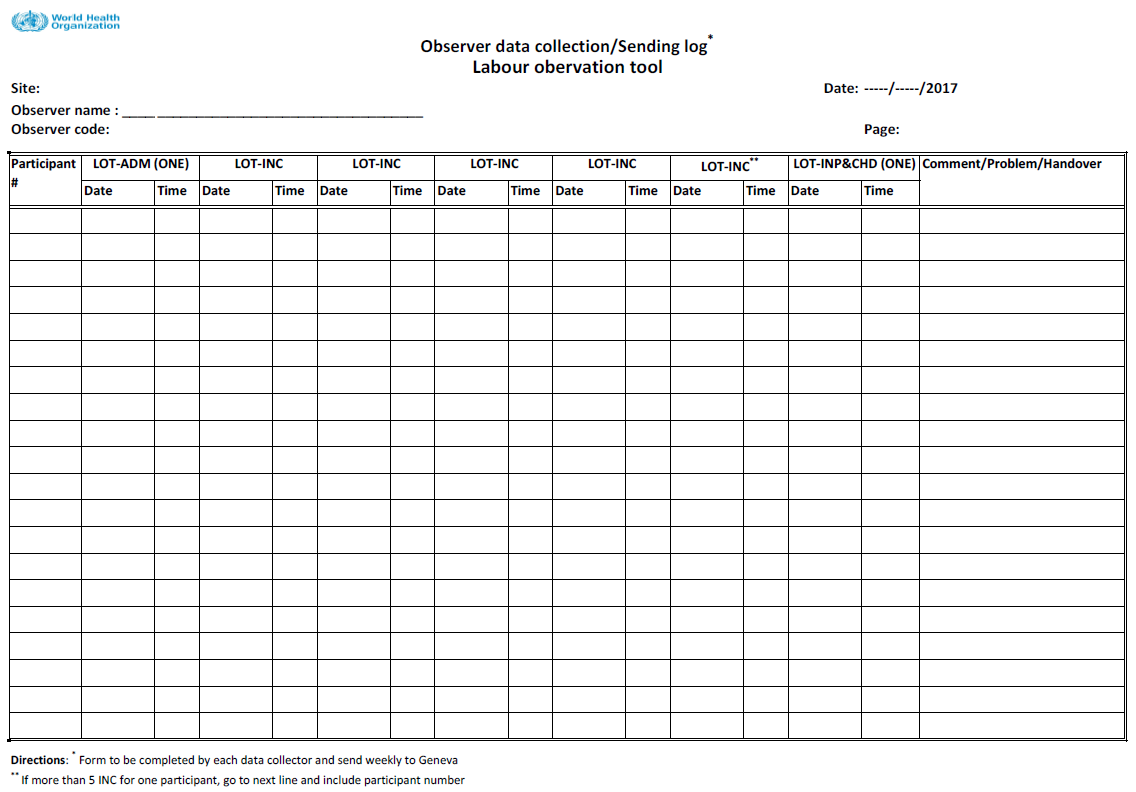

Supplement: Supplementary file 5 — Data submission log for the labor observation. This is an example of the data submission log used to record tablet-based form submission for the labor observation. (DOCX 74 kb) [file 12874_2018_603_MOESM5_ESM.docx]

**Appendix 6. Data submission log for the community survey tool**


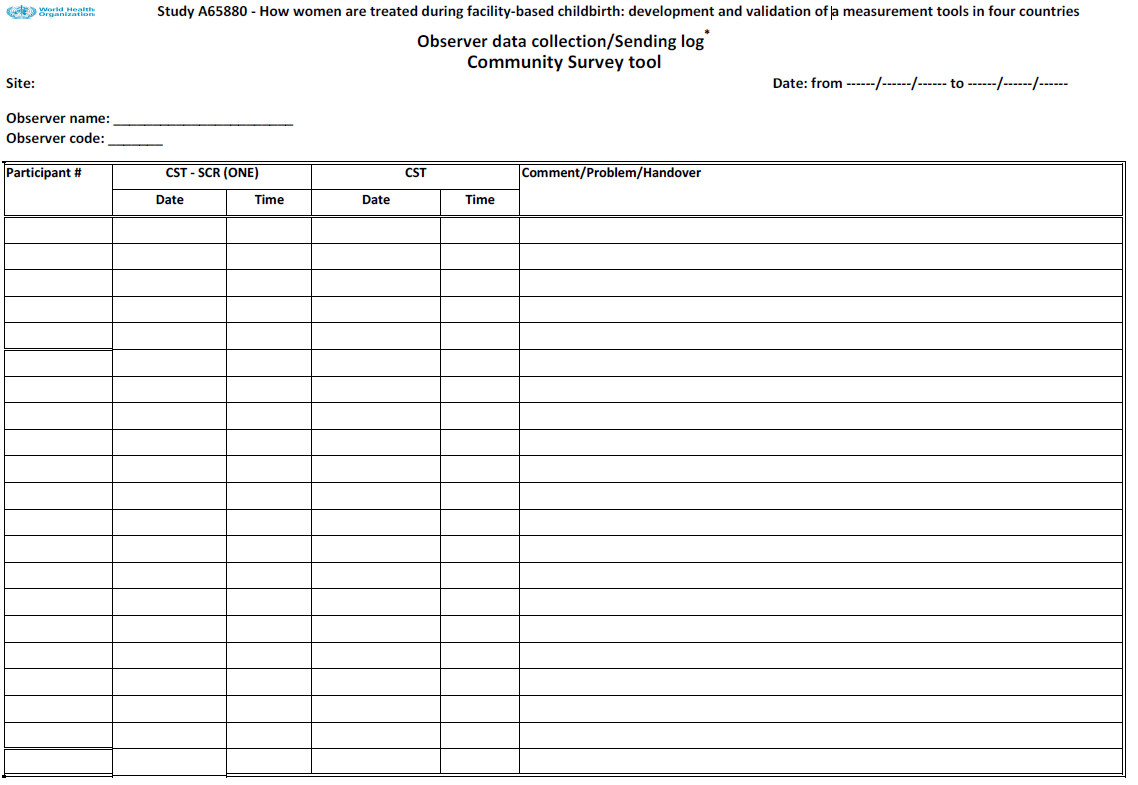

Supplement: Supplementary file 6 — Data submission log for the community survey. This is an example of the data submission log used to record tablet-based form submission for the community survey. (DOCX 65 kb) [file 12874_2018_603_MOESM6_ESM.docx]

**Appendix 7. Data collection discrepancy report**


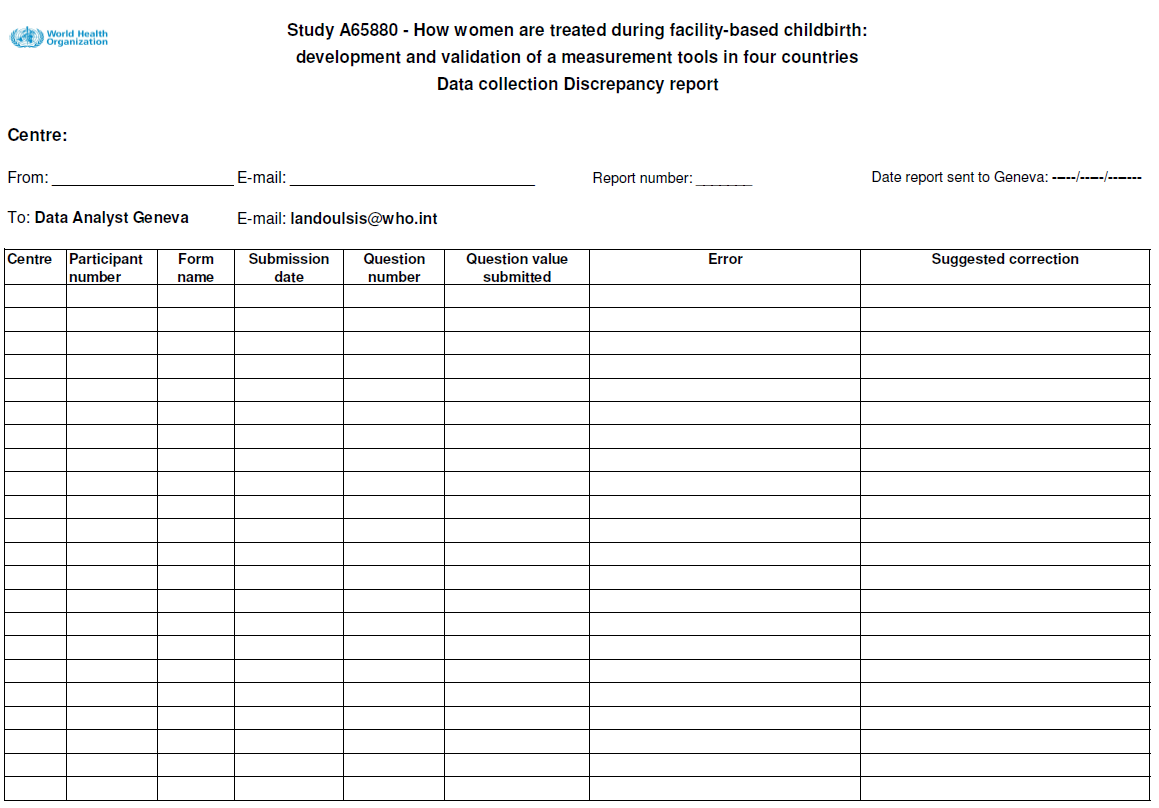

Supplement: Supplementary file 7 — Data collection discrepancy report. This is an example of the data collection discrepancy report used by the data collectors to identify and suggest corrected values for any errors on forms that were already submitted. (DOCX 71 kb) [file 12874_2018_603_MOESM7_ESM.docx]
